# *Toxoplasma gondii gra5* deletion mutant protects hosts against *Toxoplasma gondii* infection and breast tumors

**DOI:** 10.3389/fimmu.2023.1173379

**Published:** 2023-06-23

**Authors:** Min Chen, Pei Yang, Zixuan Xin, Jiating Chen, Weihao Zou, Lijuan Zhou, Lili Yang, Jiao Peng, Hongjuan Peng

**Affiliations:** Department of Pathogen Biology, Guangdong Provincial Key Laboratory of Tropical Disease Research, School of Public Health, Southern Medical University, Guangzhou, Guangdong, China

**Keywords:** *Toxoplasma gondii*, GRA5, vaccine, immune response, 4T1 tumor

## Abstract

*Toxoplasma gondii* is the causative agent of toxoplasmosis, a zoonotic disease that poses a threat to human health and a considerable loss to livestock farming. At present, clinical therapeutic drugs mainly target *T. gondii* tachyzoites and fail to eradicate bradyzoites. Developing a safe and effective vaccine against toxoplasmosis is urgent and important. Breast cancer has become a major public health problem and the therapeutic method needs to be further explored. Many similarities exist between the immune responses caused by *T. gondii* infection and the immunotherapy for cancers. *T. gondii* dense granule organelles secrete immunogenic dense granule proteins (GRAs). GRA5 is localized to the parasitophorous vacuole membrane in the tachyzoite stage and the cyst wall in the bradyzoite stage. We found that *T. gondii* ME49 *gra5* knockout strain (ME49Δ*gra5*) was avirulent and failed to form cysts but stimulated antibodies, inflammatory cytokines, and leukocytes infiltration in mice. We next investigated the protective efficacy of ME49Δ*gra5* vaccination against *T. gondii* infection and tumor development. All the immunized mice survived the challenge infection of either wild-type RH, ME49, VEG tachyzoites, or ME49 cysts. Moreover, ME49Δ*gra5* tachyzoite inoculation *in situ* attenuated the growth of murine breast tumor (4T1) in mice and prevented 4T1’s lung metastasis. ME49Δ*gra5* inoculation upregulated the levels of Th1 cytokines and tumor-infiltrating T cells in the tumor microenvironment and triggered anti-tumor responses by increasing the number of natural killer, B, and T cells, macrophages, and dendritic cells in the spleen. Collectively, these results suggested that ME49Δ*gra5* was a potent live attenuated vaccine against *T. gondii* infection and breast cancer.

## Introduction

Toxoplasmosis, a worldwide distributed opportunistic zoonosis, is caused by the intracellular protozoon *Toxoplasma gondii*. It is estimated that 30% of the world population is infected with *T. gondii.* The primary infection among the immune-competent population is subclinical and among pregnant women will result in serious damage to the fetus, and the reactivation of latent infection in immunocompromised patients can result in serious symptoms, such as encephalitis and retinochoroiditis (1). Upon active host cell entry, the fast-replicating tachyzoites form a parasitophorous vacuole (PV), in which they reside safely (2). Bradyzoites are slow-growing parasites inside a thick cyst wall and responsible for chronic infection (3). The proteins on the PV membrane (PVM) and the cyst wall are composed of the host cell membrane proteins as well as the proteins secreted by *T. gondii*, including dense granule proteins (GRAs), rhoptry proteins, and microneme proteins (MICs) which exhibit high immunogenicity (3, 4).

Ingestion of raw or undercooked meat containing *T. gondii* tissue cysts is an important mode of infection (5). Although the combination of pyrimethamine and sulfadiazine efficiently kills tachyzoites, bradyzoites are less impacted due to the low permeability of the tissue cyst wall in which they reside (6, 7). Therefore, to develop a safe and effective vaccine is urgent for the prevention of toxoplasmosis. GRA5 is reported to localize on the PVM and the cyst wall (8, 9), and the cyst interior is decreased in *gra5* mutant strain *in vitro* (10). It had been reported that the DNA vaccine encoding GRA5 elicited humoral and cellular-mediated immunity in mice, and this DNA vaccine can partially protect against *T. gondii* acute infection in BALB/c mice (11). The mice immunized with a multi-antigenic DNA vaccine encoding SAG1, SAG3, MIC4, GRA5, GRA7, AMA1, and BAG1 produced a higher level of CD3^+^, CD4^+^, and CD8^+^ cells, IL12, and IFN-γ to protect the mice against *T. gondii* infection (12).

The anti-pathogen immunity also functions in tumor inhibition. A combination of cowpea mosaic virus immunotherapy and cyclophosphamide inhibited 4T1 murine tumor growth, as well as lung metastasis (13). *T. gondii* infection induces a Th1 immune response that reduces the Lewis lung carcinoma burden (14). The nonreplicating avirulent uracil auxotroph vaccine strain (*cps*) of *T. gondii* triggers antitumor immune responses against B16F10 melanoma, ovarian cancer, and 4T1 murine cancer (15–17). An intratumoral injection of the avirulent Δ*gra17* combined with anti-programmed death ligand 1 therapy controls the growth of murine B16-F10, MC38, and LLC tumors by the synergy effects of these two therapies (18).

Immunotherapy is very important for cancer treatment and has achieved great success recently (19). Among immune cells, cytotoxic CD8^+^ T cells have main effects on prolonging the survival of cancer patients (20). T helper (T_h_) cells, also known as CD4^+^ T cells, can suppress tumor growth through secreting proinflammatory cytokines, such as IFN-γ (21). B cells produce antibodies against tumor antigens, which have been frequently found in the serum of cancer patients (22). Natural killer (NK) cells are a type of cytotoxic lymphocytes critical to the innate and adaptive immune system and kill tumor cells through lysis or inducing apoptosis (20, 23). Macrophages and dendritic cells (DCs) are innate immune cells in response to tumor antigens (24). All the above-mentioned cells are present in the tumor microenvironment (TME). In addition, many processes that occur in the TME, such as cell apoptosis and immune cell infiltration, are related to cancer immune therapy (25).

Breast cancer (BC) is the most common tumor as well as the major cause of cancer-related deaths in women all over the world (26). Triple-negative breast cancer (TNBC) is one type of BC with the cells deficient for estrogen receptor (ER), progesterone receptors (PR), and human epidermal growth factor receptor 2 (HER2) (27). Compared with other types of BC, TNBC is characterized by the most aggressive form, high recurrence, and poor prognosis and usually accompanied by increased fatality (28). Chemotherapy is the main therapeutic method for TNBC besides surgery and radiotherapy; however, chemotherapy is prone to induce drug resistance (29, 30). Because of the high expression of programmed death ligand 1, TNBC is considered a more immunogenic subtype. Therefore, immunotherapy has emerged as an important alternative in the management of this cancer (31).

In this study, we found that GRA5 was critical for *T. gondii* virulence and cysts formation in mice. ME49 with *gra5* deletion (ME49Δ*gra5)* promoted the levels of IFN-γ, IL12, and TNF-α that protected the mice from the challenge infection of different types of *T. gondii*. In addition, ME49Δ*gra5* inoculation restrained 4T1 tumor growth and metastasis. Our study supported that ME49Δ*gra5* is a promising attenuated vaccine against *T. gondii* infection and breast tumor.

## Results

### *Tg*GRA5 is critical for *T. gondii* virulence and cyst formation in mice

**Figure 1:**
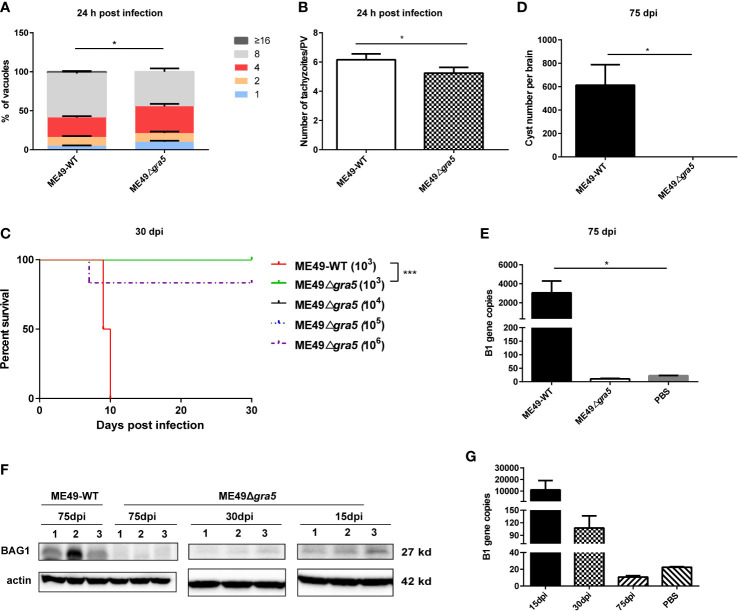
Detection of the virulence and cyst formation of ME49Δ*gra5*. **(A, B)** HFF cells were infected with ME49-WT or ME49Δ*gra5* tachyzoites at multiplicity of infection = 1 for 24 h. The number of parasites in each parasitophorous vacuole (PV) **(A)** and the average number of parasites per PV were calculated **(B)**. **(C)** Six- to 8-week-old Balb/c female mice were i.p. injected with 10^3^ ME49-WT tachyzoites or 10^3^, 10^4^, 10^5^, and 10^6^ ME49Δ*gra5* tachyzoites, respectively, and the survival of mice was monitored for 30 days (*n* = 6 mice). **(D, E)** Eight-week-old male SV129 mice were i.p. infected with 100 ME49-WT or ME49Δ*gra5* tachyzoites (*n* = 3), the mice were sacrificed, and each brain was harvested and homogenized at 75 dpi. The cysts of each brain were calculated **(D)**; the B1 copies of the brain homogenate were detected by qPCR **(E)**. **(F, G)** Six-to 8-week-old Balb/c female mice were i.p. injected with 10^3^ ME49Δ*gra5* tachyzoites. BAG1 of the brain homogenate at 15 and 30 dpi was detected by western blot **(F)**; the B1 copies of brain tissue at 15, 30 and 75 dpi were detected by qPCR **(G)**. The data are represented as mean ± SEM of three independent experiments. Statistical significance was assessed with a two-tailed unpaired Student’s *t*-test (A, B, D, E), log-rank (Mantel–Cox) test **(C)**, or Kruskal–Wallis test **(E)**. *p<0.05, ***p<0.001.

*Tg*GRA5 previously localizes to the PVM at the tachyzoite stage and later to the cyst wall at the bradyzoite stage (11). We further investigated the function of *Tg*GRA5. The *gra5* gene deletion was successfully generated using CRISPR/Cas9 technology (**Supplementary Figures S1A, B**). ME49Δ*gra5*’s infection capability was not significantly impaired compared with its parental strain ME49-WT (**Supplementary Figures S1C,D**). At 24 h post-infection, the proliferation of ME49Δ*gra5* in human foreskin fibroblasts (HFF) cells is modestly inhibited compared with ME49-WT. Each PV of ME49-WT contained approximately six tachyzoites on average, while that of ME49Δ*gra5* contained five on average (**Figures 1A, B**). In the survival assay, Balb/c mice were intraperitoneally (i.p.) infected with 10^3^ tachyzoites of ME49-WT or ME49Δ*gra5* per mouse (*n* = 6). ME49-WT parasites killed all of the mice at 10 days post-infection (dpi). However, ME49Δ*gra5* exhibited a significantly attenuated virulence; all the mice survived up to 30 dpi (**Figure 1C**). The mice challenged with ME49-WT exhibited obvious symptoms, such as body shakes and hair erecting, from 6 and 7 dpi up to their death in succession at 10 dpi. However, the mice infected with ME49Δ*gra5* showed very weak symptoms from 6 and 7 dpi up to 14 dpi. Furthermore, Balb/c mice were i.p. injected with 10^4^, 10^5^, and 10^6^ ME49Δ*gra5* tachyzoites per mouse (*n* = 6), respectively. The modified agglutination test (MAT) results confirmed that the above-mentioned mice had been successfully infected with ME49-WT or ME49Δ*gra5*, which were all positive with *T. gondii* antibody at 1:12.5 serum dilution (**Supplementary Figure S1E**). This result indicated that ME49Δ*gra5* also induced a high level of antibodies. Only one mouse infected with ME49Δ*gra5* died at the dose of 10^6^ at 7 dpi, and the survival time of the other mice exceeded 30 days (**Figure 1C**). These results indicated that GRA5 is a virulence determinant of *T. gondii* ME49.

Given that GRA5 is a cyst wall protein, we next assessed the ability of ME49Δ*gra5* to establish a chronic infection in mice. The mice were peritoneally injected with 100 tachyzoites of ME49-WT or ME49Δ*gra5*. At 75 dpi the mice were sacrificed, and the brains were harvested and homogenized. The brain homogenate smear was examined under a light microscope. We found that ME49-WT parasites formed normal cysts, with 600–800 cysts of about 50 μm in diameter on average in each brain (**Supplementary Figure S1F**); remarkably, no cyst was found in the brain homogenate of mice infected with ME49Δ*gra5* (**Figure 1D**). Moreover, neither B1 gene nor BAG1 protein was detected by qPCR or western blot in the brain homogenate of the mice infected with ME49Δ*gra5*. In contrast, both B1 gene and BAG1 protein were detected in the brains of mice infected with ME49-WT at 75 dpi (**Figure 1E**). To confirm if ME49Δ*gra5* can establish a chronic infection or not, Balb/c mice were further i.p. infected with 10^3^ ME49Δ*gra5* parasites for 15, 30, and 75 days. The mice were sacrificed, the brains were harvested and homogenized, and the B1 gene was detected by q-PCR with the brain homogenate. The B1 gene copy showed that the parasite replicated in mice at 15 dpi but decreased significantly at 30 dpi and down to undetectable levels at 75 dpi (**Figure 1G**). The BAG1 protein signal detected by western blot became weaker and weaker from 15 to 30 dpi and undetectable at 75 dpi (**Figure 1F**). These results demonstrated that GRA5 is critical for *T. gondii* virulence and cyst formation.

### The replication of ME49Δ*gra5* strain is inhibited in mice

We further investigated whether GRA5 is essential for the proliferation of *T. gondii in vivo*. Balb/c mice were i.p. injected with 10^3^ tachyzoites of ME49-WT or ME49Δ*gra5*. The weight of the mice was recorded before infection and at 7 dpi. The result showed that the weight gain of the mice infected with ME49-WT was significantly less than that of the mice infected with ME49Δ*gra5* and the phosphate-buffered saline (PBS) control group; no significant difference in body weight changes was found between the ME49Δ*gra5* infection group and the PBS control group (**Supplementary Figure S2A**). In addition, the mice were sacrificed and dissected at 7 dpi, and we found that the weight of the spleen and liver of the mice infected with ME49-WT and ME49Δ*gra5* was significantly higher than that of the PBS control group, but no significant difference in the weight of brain and lung was found among these three groups (**Supplementary Figures S2B–E**). We next explored whether the ME49Δ*gra5* strain has a replication defect in mice by detecting the parasitic load in different organs. At 7 dpi, the mice were sacrificed, and peritoneal fluid, spleen, liver, brain, and lung were harvested and subjected to qPCR to detect B1 gene copies. The parasitic load in peritoneal fluid, spleen, liver, brain, and lung in the mice infected with ME49Δ*gra5* was significantly lower than that of the mice infected with ME49-WT (**Supplementary Figures S3A–E**). These results suggested that the proliferation of *T. gondii in vivo* was significantly inhibited if *gra5* gene was deleted.

### ME49Δ*gra5* stimulates significant immune responses in mice

**Figure 2:**
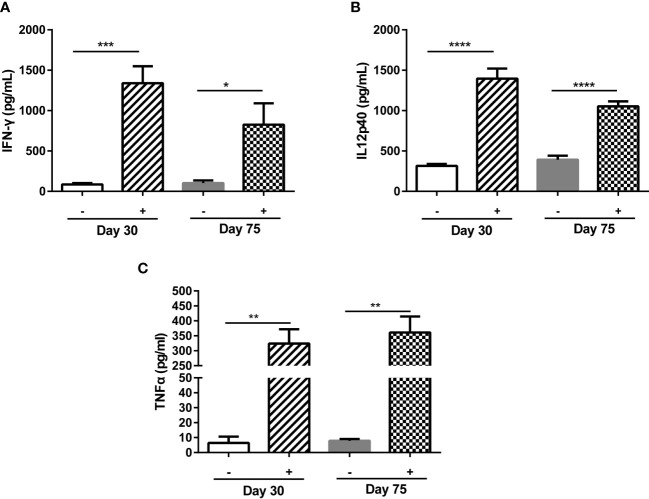
ME49Δ*gra5* parasite vaccination induced host immune responses. **(A–C)** Six-to 8-week-old female Balb/c mice were immunized with 10^3^ ME49Δ*gra5* parasites for 30 or 75 days. The serum of immunized and naïve mice (*n* = 5) was obtained and detected by ELISA for the levels of IFN-γ **(A)**, IL12p40 **(B)**, and TNF-α **(C)**. Data are represented as mean ± SEM of three independent experiments. Statistical significance was assessed with a two-tailed unpaired Student’s *t*-test. *p<0.05, **p<0.01, ***p<0.001, ****p<0.0001.

ME49Δ*gra5* stimulated a high level of antibodies in mice from 7 to 30 dpi (**Supplementary Figure S1E**). On the other hand, compared with ME49, though ME49Δ*gra5* could replicate in mice (**Figure 1G**), its multiplication ability was significantly abated *in vitro* (**Figure 1B**) and *in vivo* (**Supplementary Figure S3**) during acute infection and failed to form cysts in mice (**Figure 1D**). For these reasons, ME49Δ*gra5* would be a potent vaccine against *T. gondii* infection. According to previous reports, the efficacy of the live attenuated strain of *T. gondii* against toxoplasmosis was evaluated at 30 dpi for short-term protectivity and 75 dpi for long-term protectivity (32, 33). IFN-γ, IL12, and TNF-α are the key pro-inflammatory cytokines against *T. gondii* infection (33, 34). Therefore, we evaluated the immune protectivity of ME49Δ*gra5* strain at 30 and 75 dpi, and the levels of cytokines were measured in mice serum using ELISA. The results showed that the IFN-γ, IL12, and TNF-α levels were significantly elevated at both 30 and 75 dpi compared with the PBS control group (**Figures 2A–C**).

### ME49Δ*gra5* vaccination protects mice from tachyzoite and bradyzoite infection

**Figure 3:**
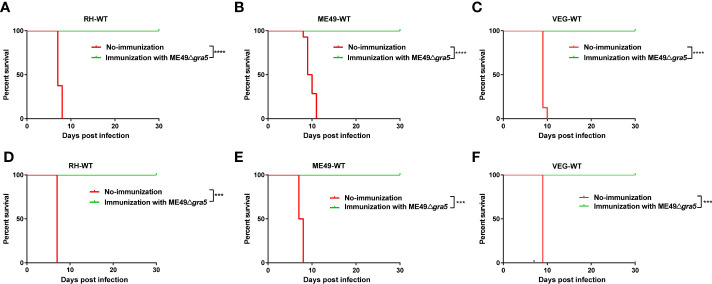
Examination of the protective effects of ME49Δ*gra5* vaccination against different types of *T. gondii*. **(A–F)** The survival curves of Balb/c mice after infection by different types of *T. gondii* strains as indicated. Six- to 8-week-old female Balb/c mice were immunized with 10^3^ ME49Δ*gra5* parasites for 30 days **(A–C)** or 75 days **(D–F)**, and then the mice were challenged with 10^3^ tachyzoites of RH-WT **(A, D)**, ME49-WT **(B, E)**, or VEG-WT **(C, F)**. All mice were monitored for 30 days, and the non-vaccinated mice were used as control (*n* = 8). Data are represented as mean ± SEM of three independent experiments. Statistical significance was assessed with log-rank (Mantel–Cox) test. ***p<0.001, ****p<0.0001.

Due to its low virulence, defect in cysts formation, and stimulation of immune responses in mice, ME49Δ*gra5* was assessed as a vaccine candidate. Balb/c mice were immunized with 10^3^ ME49Δ*gra5* tachyzoites i.p. At 30 dpi, both the vaccinated and the naïve mice were challenged with 10^3^ tachyzoites of WT RH, ME49, or VEG and were monitored for another 30 days. All non-immunized mice died within 11 days after infection, while all ME49Δ*gra5* vaccinated mice survived to the end of the experiment after infection by these three types of *T. gondii* (**Figures 3A–C**). We further detected whether ME49Δ*gra5* vaccination produced long-term immune protection against *T. gondii* challenge infection. Balb/c mice were immunized with 10^3^ ME49Δ*gra5* tachyzoites or unimmunized and then injected i.p. with 10^3^ tachyzoites of RH-WT, ME49-WT, or VEG-WT at 75 dpi and monitored for another 30 days. The survival rates of the immunized mice were 100%, while the naïve mice all died within 9 days after infection (**Figures 3D–F**).

Since ME49Δ*gra5* vaccination had a significant protective effect on *T. gondii* tachyzoite infection, we tested whether it also could effectively resist bradyzoite infection. At 30 days after the immunization with 10^3^ ME49Δ*gra5* tachyzoites, the mice were orally challenged with 20 cysts of ME49-WT. The result showed that all vaccinated mice survived the cyst challenge up to 30 dpi. However, the non-immunized mice all died within 10 days after infection (**Supplementary Figure S4**). These results demonstrate that the ME49Δ*gra5* strain can provide strong immune protection against tachyzoite and bradyzoite infections.

### ME49Δ*gra5* suppresses 4T1 tumor growth

**Figure 4:**
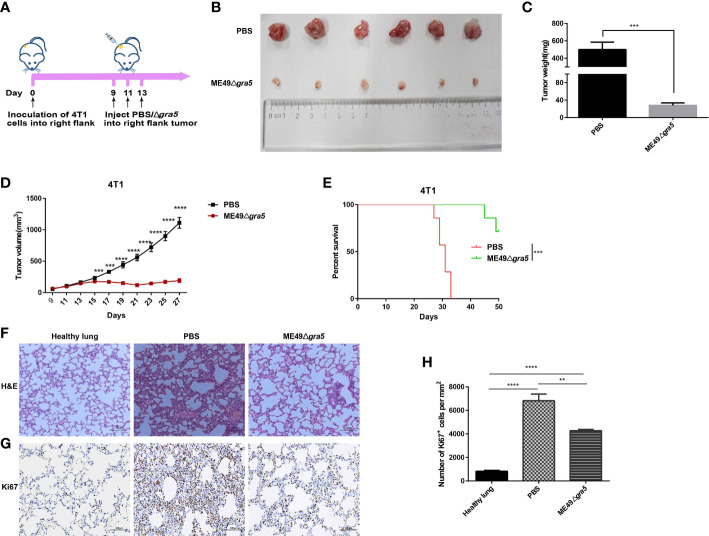
ME49Δ*gra5* tachyzoite treatment inhibited 4T1 tumor growth in mice. **(A)** Schematic diagram to show the processes of mice being inoculated with 4T1 cells and treated with ME49Δ*gra5* tachyzoites or phosphate-buffered saline (PBS). **(B–H)** Comparisons between the 4T1 tumor-bearing mice treated with ME49Δ*gra5* or PBS: tumors **(B)**, tumor size **(C)**, tumor volume **(D)**, survival curves **(E)**, lung sections stained using (H&E) staining **(F)**, and immunohistochemistry analysis of lung tissue stained with Ki67 antibody **(G)**. Abundance of Ki67-positive cells in the lung tissue **(H)**. Data are represented as mean ± SEM of three independent experiments **(A–E)**, *n* = 7; **(F–H)**, *n* = 3. Statistical significance was assessed with a two-tailed unpaired Student’s *t*-test **(D)**, log-rank (Mantel–Cox) test **(E)**, or one-way ANOVA **(H)**. **p<0.01, ***p<0.001, ****p<0.0001. Scale bar = 100 μm.

It has been previously reported that attenuated *T. gondii* inhibits tumor growth and metastasis (15, 17, 18). In our research, the avirulent ME49Δ*gra5* tachyzoites could prominently induce the production of the pro-inflammatory cytokines, such as IFN-γ, IL12, and TNF-α (**Figure 2**), which are key cytokines against tumor development (17, 18). We then continued to determine its antitumor effects with 4T1 murine breast tumor as the model. The tumor was inoculated *in situ* with ME49Δ*gra5* tachyzoites on days 9, 11, and 13 after 4T1 cell injection, with a dose of 10^5^ tachyzoites per inoculation (**Figure 4A**). The mice with an intratumoral inoculation of ME49Δ*gra5* showed remarkable tumor growth inhibition, including significantly decreased weight and size of the tumors, compared with the PBS inoculation (control) group (**Figures 4B–D**). On day 27 after tumor inoculation, the tumor volumes of the control group were sixfold larger than that of the ME49Δ*gra5* inoculation group (**Figure 4D**). ME49Δ*gra5* inoculation significantly extended the survival of the mice bearing 4T1 tumor compared with the PBS treatment (**Figure 4E**).

The 4T1 primary tumor can develop a spontaneous metastatic disease in the lungs of mice as early as 8 days after inoculation (13, 17). We continued to evaluate if ME49Δ*gra5* inoculation can inhibit 4T1 metastasis. The lung sessions with hematoxylin and eosin (H&E) staining showed that, compared with the PBS group, the infiltrating myeloid cells were significantly reduced, and the alveolar spaces were larger in the ME49Δ*gra5* inoculation group (**Figure 4F**). We also assessed 4T1 lung metastasis by using Ki67 antibody to stain the lung tissue. Less Ki67-positive cells were detected in the lungs of the mice inoculated with ME49Δ*gra5* compared with the control group (**Figures 4G, H**). Based on these results, we conclude that ME49Δ*gra5* parasite treatment inhibits 4T1 tumor growth and lung metastasis.

### ME49Δ*gra5* treatment increases tumor-infiltrating lymphocytes

**Figure 5:**
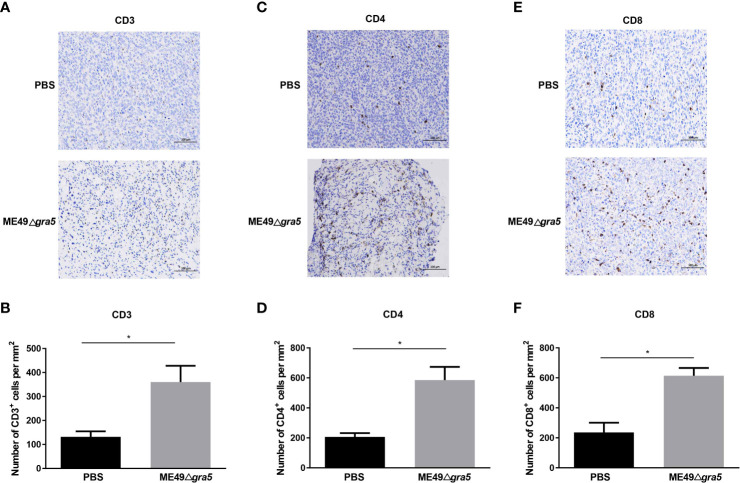
ME49Δ*gra5* tachyzoite inoculation increased the tumor-infiltrating lymphocytes in mice. **(A–F)** 4T1 tumors were harvested on day 21 after 4T1 cell inoculation and used for immunohistochemistry staining for CD3 **(A)**, CD4 **(C)**, and CD8 **(E)**. The numbers of CD3+ **(B)**, CD4+ **(D)**, and CD8+ **(F)** cells were calculated and compared between the PBS and the ME49Δ*gra5* inoculation groups. Data are represented as mean ± SEM of three independent experiments (*n* = 3). Statistical significance was assessed with a two-tailed unpaired Student’s *t*-test. *p<0.05. Scale bar=100 μm.

Cytokines secreted by immune cells play crucial roles in suppressing tumor progression. IL12 is the primary driver of Th1 differentiation and stimulates the production of IFN-γ to coordinately attack tumors (13). Therefore, we performed ELISA assay to estimate the levels of IFN-γ and IL12 in the serum and homogenized tumor lysate of the mice after ME49Δ*gra5* inoculation. We found that ME49Δ*gra5 in situ* inoculation increased the level of IFN-γ and IL12 in both mice serum and the TME compared with PBS inoculation (**Supplementary Figure S5**). Cytokines activate immune cells and induce their concentration to kill tumor cells. As expected, ME49Δ*gra5* therapy increased the population of T cells, as indicated by the increased CD3^+^ (**Figures 5A, B**), CD4^+^ (**Figures 5C, D**), and CD8^+^ cells (**Figures 5E, F**). Taken together, ME49Δ*gra5* treatment elevates the levels of antitumor cytokines and the amounts of tumor-infiltrating lymphocytes.

### ME49Δ*gra5* treatment enhances the anti-tumor immune responses of spleen

**Figure 6:**
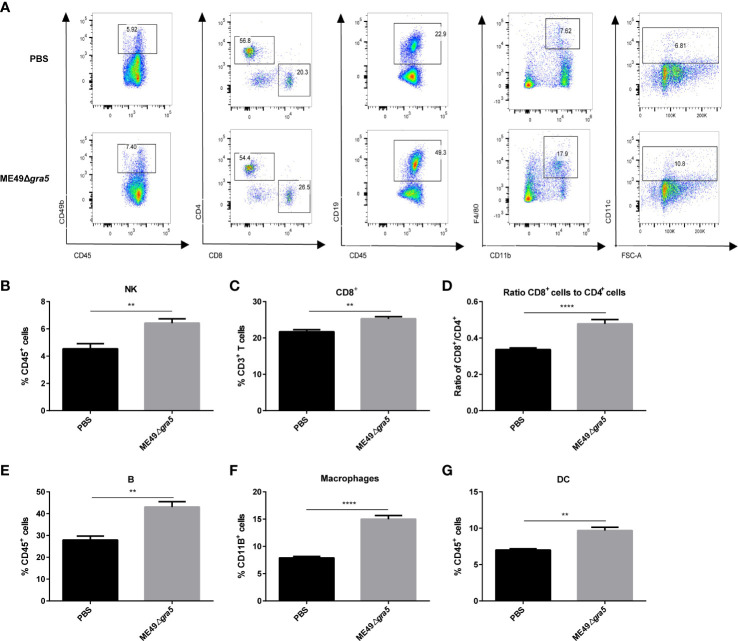
ME49Δ*gra5* therapy increased the percentages of innate and adaptive immune cells in the spleen. The mice bearing 4T1 tumors were inoculated with ME49Δ*gra5* or PBS. The spleens of all mice were collected on day 21 after inoculation, and the splenocyte was detected by flow cytometry. **(A)** Representative plots of flow cytometry data are presented in the panel. **(B–G)** Percentage of immune cells: NK cells **(B)**, CD8^+^ T cells **(C)**, B cells **(E)**, macrophages **(F)**, and DC cells **(G)**, and the ratio of CD8^+^/CD4^+^ **(D)**. Data are represented as mean ± SEM of three independent experiments (*n* = 6). Statistical significance was assessed with a two-tailed unpaired Student’s *t*-test. **p<0.01, ****p<0.0001.

We further analyzed the immune status of spleen at 21 dpi. The innate immune cells, CD49b, F4/80, and CD11c representing NK cells, macrophages, and DCs, respectively, were remarkably increased in ME49Δ*gra5*-treated mice (**Figures 6A, B, F, G**). CD8^+^ T cells are the major cell type mediating anti-tumor effects, and the CD8^+^/CD4^+^ ratio indicates the amount of CD8^+^ T cells of specific anti-tumor immunity (35). We found that the amount of CD8^+^ T cells and the ratio of CD8^+^/CD4^+^ were significantly elevated in ME49Δ*gra5* treated*-*mice (**Figures 6A, C, D**). The production of CD4^+^ T follicular helper cells requires B cells that recognize tumor neoantigens, and their collaboration promotes anti-tumor CD8^+^ T cells responses (36). Concordantly, the quantity of B cells marked by CD19 (a marker of B cells), was significantly increased in the ME49Δ*gra5* therapy group (**Figures 6A, E**). The above-mentioned results were consistent with the immune responses in TME (**Figure 5**).

### ME49Δ*gra5* reduces the burden of non-injected distant tumors

**Figure 7:**
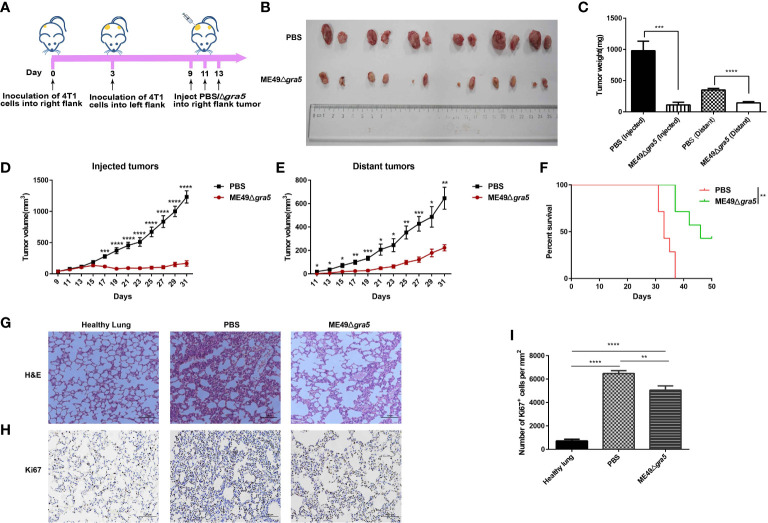
ME49Δ*gra5* parasite therapy suppressed the bilateral 4T1 tumor growth. **(A)** Schematic diagram to show the establishment processes of bilateral 4T1 mice model and treatment with ME49Δ*gra5* or phosphate-buffered saline (PBS). **(B–I)** Comparison of the bilateral 4T1 tumor-bearing mice treated with ME49Δ*gra5* or PBS: bilateral 4T1 tumors **(B)**, size of bilateral 4T1 tumors **(C)**, volumes of the injected tumors **(D)**, volumes of the distant tumors **(E)**, survival curves **(F)**, lung sections stained with H&E **(G)**, immunohistochemistry staining of lung tissue with Ki67 antibody **(H)**, and the number of Ki67-positive cells in lung tissues **(I)**. Data are represented as mean ± SEM of three independent experiments (A–F, *n* = 7; G–I, *n* = 3). Statistical significance was assessed with a two-tailed unpaired Student’s *t*-test **(D, E)**, log-rank (Mantel–Cox) test **(F)**, or one-way ANOVA **(I)**. *p<0.05, **p<0.01, ***p<0.001, ****p<0.0001. Scale bar = 100 μm.

To determine whether ME49Δ*gra5* can activate systemic antitumor immunity, we constructed a bilateral 4T1 tumor mouse model. 4T1 cells were injected into both flanks of mice, followed by inoculating ME49Δ*gra5* tachyzoites *in situ* to the right flank tumor on days 9, 11, and 13 after 4T1 cell inoculation (**Figure 7A**). We found that ME49Δ*gra5* treatment inhibited the growth of the injected (right flank) and the distant (left flank) tumors (**Figures 7B–E**). Consequently, the survival of 4T1-bearing mice in the ME49Δ*gra5* therapy group was much longer than that in the PBS treatment group (**Figure 7F**). The lung sections were stained with H&E. The results revealed fewer infiltrating myeloid cells and larger alveoli spaces in the ME49Δ*gra5* treatment group than those in the PBS treatment group (**Figure 7G**). Staining of lung tissue with Ki67 to detect 4T1 metastasis by immunohistochemistry revealed a weaker lung metastasis with a lower expression of Ki67 in ME49Δ*gra5*-inoculated mice than that in the PBS treatment mice (**Figures 7H, I**).

**Figure 8:**
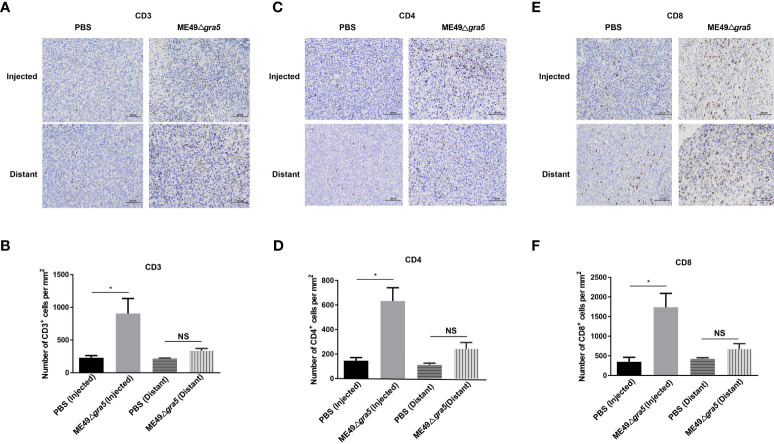
Detection of the immune status of the ME49Δ*gra5* injected and distant tumors. **(A–F)** The bilateral 4T1 tumors were harvested on day 23 after 4T1 cell inoculation and used for immunohistochemistry staining for CD3 **(A)**, CD4 **(C)**, and CD8 **(E)**. The numbers of CD3+ **(B)**, CD4+ **(D)**, and CD8+ **(F)** cells were calculated and compared between the phosphate-buffered saline and the ME49-Δ*gra5* inoculation groups for the injected and distant tumors. Data are represented as mean ± SEM of three independent experiments (*n* = 3). Statistical significance was assessed with a two-tailed unpaired Student’s *t*-test. *p<0.05. Scale bar = 100 μm.

To examine the immune system response and the immune status of the distant tumors, we first identified the cytokines that may suppress tumor growth. Mice serum, parasite-inoculated tumor, and distant tumor were collected, homogenized, and subjected to cytokine detection with ELISA. The levels of IFN-γ and IL12 were increased both in the parasite-inoculated tumor and the distant tumor of ME49Δ*gra5-*treated mice and had no significant difference between these two TMEs (**Supplementary Figures S6A, B**). The serum IFN-γ and IL12 showed the same increment in ME49Δ*gra5* therapy and the PBS treatment mice (**Supplementary Figures S6C, D**). In addition, we detected immune cells in the parasite-inoculated and the distant tumors. The population of the innate immune cells and T cells, such as CD3^+^ (**Figures 8A, B**), CD4^+^ (**Figures 8C, D**), and CD8^+^ (**Figures 8E, F**), was significantly increased in the TMEs of ME49Δ*gra5-*injected mice compared with the PBS-treated mice.

## Discussion

GRA proteins are key factors in regulating cyst formation and maintenance, and so far, more than 60 GRA proteins have been identified (37). The transmembrane domain-containing GRA proteins, including GRA5, are translocated across the endoplasmic reticulum (ER) membrane into the lumen and exported from the ER, then transported from the Golgi to the dense granules, and ultimately secreted out of the parasite (38, 39). During the early differentiation of the tachyzoite-to-bradyzoite transition, the intro-vacuole network (IVN)-associated GRA proteins, including GRA1/2/4/6/9/12, translocate from the IVN to the cyst wall (3, 4). Many bradyzoite-specific GRAs have been reported, such as MCP3 knockouts form smaller cysts, without other obvious defects (3). CST2 knockouts show an attenuated virulence, and no cyst is detected in mice (3). GRA55 knockouts exhibit a lower cyst burden in mice (40). In our research, in the brain homogenate of the mice infected with ME49Δ*gra5*, no cyst was detected at 75 dpi (**Figure 1D**); the BAG1 protein band was detected weaker and weaker by WB at 15 and 30 dpi and became undetectable at 75 dpi (**Figure 1F**), but the B1 gene which is present in both tachyzoites and bradyzoites replicated significantly at 15 dpi, compared with 7 and 30 dpi, and then became undetectable at 75 dpi (**Figure 1G**). It may be due to the reason that, though ME49Δ*gra5* can be transformed into bradyzoites, the deficiency of the cyst wall protein GRA5 resulted in a defect of cyst wall formation. Gradually, the bradyzoite of *T. gondii* was eliminated by host immunity.

The attenuated α-*amy* or *gra9* knockout strains can serve as vaccines against acute and chronic *T. gondii* infection (34, 41). However, as a few cysts are still formed by these attenuated strains, the safety is challenged. In this study, no cyst was observed in ME49Δ*gra5* infection. Meanwhile, it induced a long-term immune response in mice. Several important cytokines, including IFN-γ, IL12, and TNF-α, were induced to secret at high levels in Balb/c mice after vaccination with ME49Δ*gra5* tachyzoites. ME49Δ*gra5* immunization to mice achieved 100% protection against the challenge infection with the tachyzoites of RH, ME49, and VEG strains and ME49 cysts. Therefore, ME49Δ*gra5* vaccination is effective against a broad spectrum of *T. gondii* strains infection.

The immune response is involved in the occurrence and development of tumors, including non-specific immunity and specific immunity, of which specific immunity is divided into cellular immunity and humoral immunity. The differentiated macrophages have two phenotypes: one is classically activated macrophages (M1) and the other is alternatively activated macrophages (M2) (42). M1 macrophages can be activated by IFN-γ to produce pro-inflammatory cytokines with anti-tumor effects, while M2 macrophages promote the proliferation, invasion, and metastasis of tumor cells (43). NK cells are cytotoxic innate lymphoid cells which are able to eliminate malignant cells and limit tumor metastases through secreting IFN-γ (44). Cellular immunity is the main way of the immune responses against tumors, including CD4^+^ T cells and CD8^+^ T cells (45). Cytotoxic CD8^+^ T cells, activated by antigen-presenting cells, are the most vital in anti-cancer immune responses and constitute the backbone of cancer immunotherapy (46). CD4^+^ T cells can directly kill the tumor cells by producing a high level of IFN-γ. Moreover, it can enhance anti-tumor cytotoxic T-lymphocyte (CTL) response by promoting clonal replication at the tumor site and acting as APC for CTLs to preferentially generate immune memory cells (47). In addition to IFN-γ, IL12 also plays an essential role in anti-tumor immunity. IL12 is beneficial to cytotoxic lymphocyte maturation and is a stimulatory factor of NK cells (48, 49). In our research, the levels of IFN-γ and IL12 were much higher both in the TME and serum of the 4T1-bearing mice immunized with ME49Δ*gra5* tachyzoites. Furthermore, ME49Δ*gra5 in situ* inoculation also elevated the levels of IFN-γ and IL12 in distant TME. The immune cells that process and present tumor antigens, such as DCs, and the cells killing tumors, such as NK cells, CD4^+^ T cells, and CD8^+^ T cells, were all significantly increased in the spleens of ME49Δ*gra5*-vaccinated mice. Furthermore, the number of infiltrated CD3^+^, CD4^+^, and CD8^+^ T cells was also significantly elevated in the TME with ME49Δ*gra5* injection compared with PBS injection, but no significant difference was found with these infiltrated cells in the TME of the distant 4T1 tumor and the PBS-injected tumor. Moreover, T-regulatory cells (Treg) and myeloid-derived suppressor cells (MDSC) are the main immunosuppressive cells (47, 50). Antigen-experienced CD4^+^ T cells can develop into Tregs by expressing CD25 or Foxp3 surface markers (47)—for example, using anti-CD25 monoclonal antibodies to enhance the anti-tumor immunity of mice (51, 52). After treatment with an intratumoral injection of oncolytic virus, the Treg cells significantly decrease (13, 35). MDSC are dominated by the surface molecule CD11b, which can inhibit the activation, transport, and immune response of T cells (53). In this study, the changes of Treg and MDSC in tumor-bearing mice after ME49Δ*gra5* inoculation require further investigation. These results indicated that these immune cells have an important role in restraining tumor growth at the site with ME49Δ*gra5* infection. The antibodies and cytokines induced by this parasite infection are key factors in the inhibition of *T. gondii* tachyzoites and tumor cells. An intratumoral injection of the avirulent Δ*gra17* mutant showed a similar mechanism (18). However, Δ*gra17* mutant formed few brain cysts in infected mice (54). In our study, we demonstrated that 4T1 intratumor injection with ME49Δ*gra5* suppressed the growth of both the injected tumor and the non-injected distant tumor and prevented 4T1’s lung metastasis. Moreover, ME49Δ*gra5* parasites cannot form brain cysts, which implied that it may be safer than the Δ*gra17* mutant. Taken together, ME49Δ*gra5* parasites inhibited tumor growth through immune modulation.

**Figure 9:**
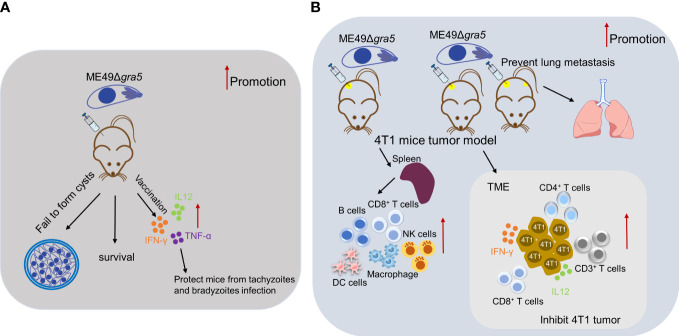
Summary for the potential of ME49Δ*gra5* as an attenuated live vaccine against *T. gondii* infection and immuno-therapeutic agent against 4T1 tumor. **(A)** ME49Δ*gra5* vaccination against *T. gondii* infection. **(B)** ME49Δ*gra5* inoculation against 4T1 tumor.

In summary, as shown in **Figure 9**, our results demonstrated that GRA5 is critical for *T. gondii*’s virulence and cyst formation. ME49Δ*gra5* tachyzoite vaccination triggered the generation of pro-inflammatory factors to protect the mice from the challenge infection of three types of *T. gondii*, RH, ME49, and VEG, and this protection was long-lasting. Moreover, the intratumoral injection of ME49Δ*gra5* showed a high efficacy against the growth of the injected and distant 4T1 tumors as well as lung metastasis. ME49Δ*gra5* inoculation promoted the production of IFN-γ and IL12 and increased the innate and adaptive immune cells of the spleen and tumor-infiltrating lymphocytes. ME49Δ*gra5* was shown to be a promising vaccine against *T. gondii* infection and a potential immunotherapeutic agent against tumors.

## Materials and methods

### Animal and animal ethics

Specific pathogen-free (SPF) female Balb/c mice at 6–8 weeks old and SPF male SV129 mice at 8 weeks old were purchased from the Laboratory Animal Centre and raised in an SPF laboratory in the university. All animal experiments were performed following the guidelines for laboratory animal manipulation (permit number L2019155).

### Cells and parasites

The HFF [American Type Culture Collection (ATCC), USA] cells were cultured in Dulbecco’s modified Eagle’s medium (DMEM, Gibco, USA), and 4T1 cells (ATCC) were kept in our lab and were cultured in Roswell Park Memorial Institute-1640 (RPMI-1640, Gibco) supplemented with 10% fetal bovine serum (FBS, Gibco) and 1% penicillin–streptomycin (Thermo Fisher Scientific) at 37°C and 5% CO_2_. RH-WT, ME49-WT, VEG-WT, and ME49Δ*gra5* tachyzoites were maintained in HFF cells cultured in DMEM supplemented with 1% FBS and penicillin–streptomycin.

### Antibodies

The antibody used for western blot—rabbit monoclonal anti-β-actin—was purchased from Cell Signaling Technology, and mouse monoclonal anti-BAG1 was raised in our lab. For the immunofluorescence assay, mouse monoclonal anti-SAG1, goat anti-mouse IgG H&L (Alexa Fluor^®^ 488), and goat anti-mouse IgG H&L (Alexa Fluor^®^ 594) were purchased from Abcam (United Kingdom). The antibodies used for immunohistochemistry—anti-Ki67 rabbit polyclonal antibody, anti-CD3 rabbit polyclonal antibody, anti-CD4 rabbit polyclonal antibody, and anti-CD8 rabbit polyclonal antibody—were purchased from Servicebio (China).

### Construction of *T. gondii* ME49Δ*gra5* strain

The *gra5* knockout ME49 strain (ME49Δ*gra5*) was constructed by CRISPR-Cas9 technology (**Supplementary Figure S1** in supplemental data). Using the primers listed in **Supplementary Table S1**, the homologous fragment was amplified using the ME49-WT genome as a template, and the pSAG1::CAS9-U6::sgUPRT vector was used as a template to construct the pSAG1::CAS9-U6::sgGRA5 plasmid. The homologous template and the sgRNA CRISPR plasmid were co-transfected to ME49-WT parasites, and the parasites were screened with the DMEM complete medium containing 3 μM pyrimethamine (Sigma, Germany). The stable lines of gene knockout parasites selected by limited dilution were confirmed by PCR. The sequences of the primers used in generation are shown in **Supplementary Table S1**.

### Immunofluorescence assay

For the invasion assay, the HFF cells were fixed with 4% paraformaldehyde for 5 min at room temperature (RT) and then blocked with 10% bovine serum albumin (BSA) in PBS for 1 h at 37°C, followed by incubation in the mouse anti-TgSAG1 monoclonal primary antibody (Abcam; 1:800 diluted in PBS) at 4°C overnight. The cells were then washed for three times with PBS, after that, incubated with the Alexa Fluor^®^ 488 conjugated goat anti-mouse IgG secondary antibody (Invitrogen, United States; 1:2,000 diluted in PBS) for 1 h at 37°C. Subsequently, the cells were permeabilized with 0.5% Triton X-100 (DingGuo, China) for 10 min at RT and blocked with 10% BSA in PBS for 1 h at 37°C. The cells were incubated again with the mouse anti-*Tg*SAG1 monoclonal primary antibody (Abcam) at 4°C overnight, followed by incubation with the Alexa Fluor^®^ 594 conjugated goat anti-mouse IgG secondary antibody (Invitrogen) for 1 h at 37°C. After washing for three times with PBS, the coverslips were taken out and rinsed with double-distilled water (ddH_2_O) and mounted with DAPI mounting oil (Southern Biotech, United States).

For the proliferation assay, the cells were fixed with 4% paraformaldehyde for 10 min at RT, then permeabilized with 0.5% Triton X-100 for 10 min at RT, and blocked with 10% BSA in PBS for 1 h at 37°C. The cells were incubated with the mouse anti-*Tg*SAG1 monoclonal primary antibody (Abcam) at 4°C overnight, followed by incubation with the Alexa Fluor^®^ 488 conjugated goat anti-mouse IgG secondary antibody (Invitrogen) for 1 h at 37°C. After that, the coverslips were washed with PBS, rinsed with ddH_2_O, and mounted with DAPI mounting oil (Southern Biotech).

### Comparison of the acute virulence (parasite’s invasion, proliferation, and host survival) for ME49 and ME49Δ*gra5*

HFF cells were seeded in 12-well plates, grown to 100% confluence, infected with ME49-WT or ME49Δ*gra5* strains (three wells for each group) at a multiplicity of infection (MOI) of 3 for the invasion assay and at MOI of 1 for the proliferation assay, and then washed with PBS for three times after 1 h. For the invasion assay, the cells were subjected to immunofluorescence assay (IFA) immediately. For the proliferation assay, the cells were further cultured for 24 h and then subjected to IFA after washing for three times with PBS. The number of parasites in each PV and the average number of parasites per PV were calculated.

For the survival assay, 6–8-week-old Balb/c female mice were i.p. injected with 10^3^ ME49-WT tachyzoites or 10^3^, 10^4^, 10^5^, and 10^6^ ME49Δ*gra5* tachyzoites, respectively, and the survival of the mice was monitored for 30 days (*n* = 6 mice). The sera of the infected mice were collected from the tails at 7 dpi for ME49-WT infection or at 7, 14, 21, and 30 dpi for the ME49Δ*gra5* infection and subjected to antibody detection by MAT (55). The sera of the ME49-WT chronically infected mice were used as the positive control.

### Cyst formation assay for ME49-WT and ME49Δ*gra5*

SV129 male mice at 8 weeks old were i.p. challenged with 100 fresh tachyzoites of ME49-WT or ME49Δ*gra5* (three mice per group). After 75 dpi, the mice were sacrificed, and the brains were harvested, homogenized, and fixed. The brain homogenate was smeared on slides and stained with FITC-conjugated dolichos biflorus agglutinin antibody (Vector Laboratories, USA), and then the cysts were calculated under a fluorescence microscope. The brain homogenate was subjected to B1 gene copy detection with q-PCR. The primers are provided in **Supplementary Table S1**.

We further analyzed the cyst formation for ME49Δ*gra5* at different times after infection. Balb/c female mice at 6–8 weeks old were i.p. injected with 10^3^ ME49Δ*gra5* tachyzoites (*n* = 9), and the mice i.p. infected with 100 ME49-WT tachyzoites for 75 days were used as the positive control (*n* = 3). The mice were sacrificed, and the brains were harvested and homogenized. The BAG1 protein in each brain homogenate were detected by western blot at 15, 30, and 75 dpi. The B1 copies in each brain homogenate were detected by qPCR at 15, 30, and 75 dpi.

### Evaluation of the protection of ME49Δ*gra5* against the challenge infection of RH, ME49, and VEG

Female Balb/c mice at 6–8 weeks old were infected with 10^3^ freshly egressed tachyzoites of ME49Δ*gra5* for 30 or 75 days. On days 30 and 75 dpi, tail blood of immunized mice and naïve mice was obtained for detection, and then these mice were i.p. injected with 10^3^ freshly egressed tachyzoites of RH-WT, ME49-WT, or VEG-WT (eight mice per group). Moreover, two other groups of immunized mice and naïve mice were orally infected with 20 fresh brain cysts of ME49 to assess the efficacy against bradyzoite infection (five mice per group). These mice were monitored daily for another 30 days.

### Evaluation of the parasitic burden *in vivo*

Female Balb/c mice at 6–8 weeks old were infected with 10^3^ freshly egressed tachyzoites of ME49-WT or ME49Δ*gra5* by i.p. injection (five mice per group). Before infection and at 7 dpi, each mouse was weighed, and the weight change was recorded. At 7 dpi, the mice were sacrificed and dissected. The murine brains, livers, spleens, and lungs were collected and weighed, respectively. In total, 10 mg spleen, 25 mg brain, lung, liver, and 1 ml peritoneal fluid were collected and used for genomic DNA extraction with DNeasy Blood and Tissue Kit (Qiagen, Germany). The parasitic burden from each sample was determined by detecting the B1 gene copies with qPCR using Hieff^®^ qPCR SYBR^®^ Green Master Mix (Low Rox Plus; Yeasen, China).

### Construction of a mouse tumor model and treatment of mice with ME49Δ*gra5*

4T1 murine breast tumor cells were cultured, harvested, and then resuspended in RPMI-1640 medium at a concentration of 1 × 10^6^ cells/ml. For the unilateral 4T1 mice tumor model, 5- to 6-week-old Balb/c female mice were subcutaneously (s.c.) injected on the right flank with 100 μl of the 4T1 cell mixture (10^5^ cells per mouse), with seven mice per group. Tumors were allowed to grow for 9 days, and then 50 μl PBS or 1 × 10^5^ ME49Δ*gra5* parasites were intratumorally injected on days 9, 11, and 13 after 4T1 cell injection. Next, the tumor size in each mouse was recorded every 2 days, and mice survival was also monitored. At 21 dpi, the mice were sacrificed, and the tumors were taken out and photographed. For the bilateral tumor model, mice were s.c. injected on the right flank with 100 μl of the 4T1 cell mixture (10^5^ cells per mouse), with seven mice per group. At 3 days later, the mice were s.c. inoculated on the left flank with the same amount of 4T1 cells. After 6 days (tumors were allowed to grow for 6 days on the left flank and 9 days on the right flank), 50 μl PBS or 1 × 10^5^ ME49Δ*gra5* parasites in 50 ul PBS were injected into the right flank tumor. Tumor size and mice survival were measured every 2 days. At 23 dpi, the mice were sacrificed, and the tumors were taken out and photographed.

### Cytokine assay

Mice serum and tumor tissue were collected to detect the level of IFN-γ and IL12p40 using ELISA kits (Multi Sciences, China) based on the manufacturer’s recommendations. Mice serum was collected from the naïve mice or ME49Δ*gra5*-immunized mice at 30 or 75 dpi and the tumor-bearing mice treated with PBS or ME49Δ*gra5* tachyzoites as described above. For tumor tissue, 25 mg of tumor tissue was weighed and ground with 500 μl pre-cooled PBS to a single-cell suspension. The suspension was stored at -80°C for 30 min, then taken out, and dissolved completely in a water bath at 37°C. The suspension was mixed up and down and placed back at -80°C. After the freezing–thaw cycle was repeated for three times, the suspension was centrifuged at 4°C, 5,000 × *g*, for 5 min, and the supernatant was taken for ELISA detection.

### Flow cytometry

The mice were inoculated with 4T1 cells and were treated with ME49Δ*gra5* parasites or PBS as described above. At 21 dpi, six mice from each group were euthanized to harvest the spleens which were homogenized and filtered to get the spleen single-cell suspension, and red blood cells were lysed with erythrocyte lysis buffer. The concentration of splenocytes was adjusted to 1 × 10^6^ cells/ml with PBS after washing with RPMI-1640 medium twice. Then, the single-cell suspensions were incubated with the antibodies (Univ, China) of panel 1—percp-cy5.5 anti-mouse CD45, BV510 anti-mouse CD3e, APC anti-mouse CD4, FITC anti-mouse CD8a, PE-cy7 anti-mouse CD19, and PE anti-mouse CD49b—and panel 2—percp-cy5.5 anti-mouse CD45, PE anti-mouse F4/80, FITC anti-mouse CD11b, and APC anti-mouse CD11c—at 4°C for 30 min following the manufacturer’s guidance. The analyses of splenocytes were performed with fluorescence profiles using a FACScan flow cytometer (BD Biosciences, USA).

### Hematoxylin and eosin staining

The lungs were cut into 4-μm sections. The sections were dewaxed and stained with hematoxylin solution for 3–5 min, followed by 85% ethanol and 95% ethanol dehydration. Finally, the sections were stained with eosin dye for 5 min and dehydrated. The samples were observed, and photos were captured under a light microscope, with the nucleus stained in blue and the cytoplasm stained in red.

### Immunohistochemistry

The lungs or tumors were cut into 4-μm sections. The sections were dewaxed in xylene I and II for 15 min, dehydrated in 100%, 85%, and 75% alcohol for 5 min each, and rinsed with ddH_2_O. The samples were placed in citric acid (pH 6.0) for antigen repair. After that, the sections were put into 3% hydrogen peroxide for 25 min at RT in the dark and blocked with 3% BSA in PBS for 30 min at RT, followed by incubating with the primary antibodies (Servicebio) at 4°C overnight. Next, the slides were washed with PBS for 5 min with gentle shaking for three times and then incubated with the goat anti-rabbit IgG H&L (HRP) antibody for 50 min at RT. 3,3-N-Diaminobenzidine tertrahydrochloride (DAB) was used for staining, and hematoxylin was used for counter-staining of the nuclei. Then, the sections were dehydrated and mounted for observation, and photos were captured under a light microscope, with the nucleus stained blue and the DAB-positive stain in brownish yellow.

### Statistical analysis

SPSS20 statistical software was used to analyze the data. The analysis of the difference between the two groups was conducted by unpaired two-tailed Student’s *t*-test. One-way ANOVA or Kruskal–Wallis test was performed to compare the difference among more than two groups. The survival of mice was compared using log-rank (Mantel–Cox) test. The statistical significance was defined as *p* < 0.05*.

## Data availability statement

The raw data supporting the conclusions of this article will be made available by the authors, without undue reservation.

## Ethics statement

The animal study was reviewed and approved by Experimental Animal Care and Use Committee of Southern Medical University. Written informed consent was obtained from the owners for the participation of their animals in this study.

## Author contributions

MC and PY performed the experiments, analyzed the data, and wrote the manuscript. ZXX performed qPCR assays. JTC and WHZ performed the western blot assay. LJZ, LLY, and JP performed the flow cytometry. HJP performed study conception and designing, supervision of the research group, funding support, and drafting of the manuscript. All authors contributed to the article and approved the submitted version.
