# Supplementary material for: Toxoplasma gondii gra5 deletion mutant protects hosts against Toxoplasma gondii infection and breast tumors
Source: Front Immunol. 2023 Jun 23;14:1173379. doi: 10.3389/fimmu.2023.1173379 (PMC10327641; doi:10.3389/fimmu.2023.1173379)
Supplement: Supplementary file 1 [file DataSheet_1.zip › supplementary information/supplementary information.docx]

***Toxoplasma gondii gra5* deletion mutant Protects Hosts Against *Toxoplasma gondii* Infection and Breast Tumors**

Min Chen^#^, Pei Yang^#^, Zixuan Xin, Jiating Chen, Weihao Zou, Lijuan Zhou, Lili Yang, Jiao Peng, Hongjuan Peng*

Department of Pathogen Biology, Guangdong Provincial Key Laboratory of Tropical Disease Research, School of Public Health, Southern Medical University, Guangzhou, Guangdong Province, 510515, P. R. China.

*Correspondence to Hongjuan Peng, floriapeng@hotmail.com

#These authors contributed equally

**Table S1**

Table S1. Gene names and sequences of primers used in this study

| Gene |  | Sequences |
| --- | --- | --- |
| B1-qPCR | Forward | 5' -GGAACTGCATCCGTTCATG-3' |
|  | Reverse | 5' -TCTTTAAAGCGTTCGTGGTC-3' |
| *gra5*-5UTR | Forward | 5' -TCGACGGTATCGATAAGCTTTTGCTGCAAAGTGCCCCCCT-3' |
|  | Reverse | 5' -GGCGAAGCTTACAGTGAACCATACACACAGTTAG-3' |
| DHFR | Forward | 5' - GGTTCACTGTAAGCTTCGCCAGGCTGTAAATC-3' |
|  | Reverse | 5' -AACACAGTGCCAGGAATTCATCCTGCAAGTGC-3' |
| *gra5*-3UTR | Forward | 5' - TGAATTCCTGGCACTGTGTTGCTCGGCTCTTTG -3' |
|  | Reverse | 5' - GCTCTAGAACTAGTGGATCCGAGTTACGCGTACATAGACC-3' |
| *gra5-*KO1 | Forward | 5' -CAACTCCACGTCCTGGTGTCC-3' |
|  | Reverse | 5' -TCGCTGGTAGTCCCAACTGAAT-3' |
| *gra5-*KO2 | Forward | 5' -ATGTGGCATTTCACACAGTCTC-3' |
|  | Reverse | 5' -GTTCGCAGCGAACTACGGTGAA-3' |
| *gra5-*KO3 | Forward | 5' -GATAAAGGTCCTGCCAGGTTG-3' |
|  | Reverse | 5' -GCTCTGACAATGACGTCTGCC-3' |
| *gra5* sgRNA |  | ACGCCCACAAAAATTAAAGC |

Figure S1


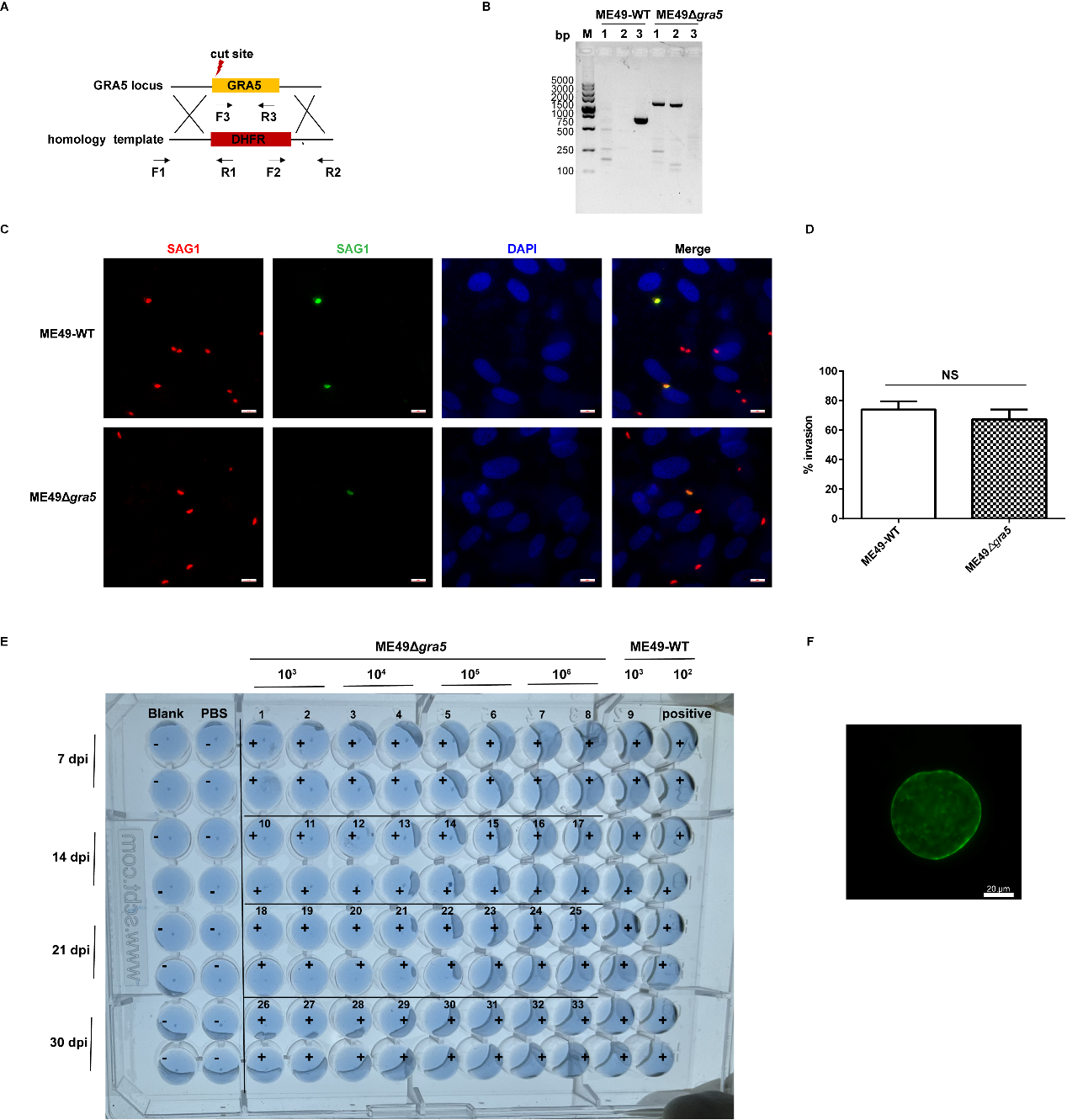


**Figure S1. Detection of the invasion efficiency of ME49∆*gra5*.** A. Schematic diagram for construction of ME49∆*gra5* by CRISPR/Cas9. B. PCR identification of ME49∆*gra5* colony. Lane 1-3: Three pairs of primers as indicated in (A) were used, and the genomic DNA of the indicated strain was used as the template. C-D. HFF cells were infected with ME49-WT or ME49∆*gra5* strains for 1 h (MOI=3). The extracellular tachyzoites were shown in green and the total tachyzoites were show in red (C); the infection rates were calculated and compared between the indicated infection groups (D). E. The sera of mice infected with10^3^ ME49-WT tachyzoites at 7 dpi, or 10^3^, 10^4^, 10^5^, and 10^6^ ME49∆*gra5* tachyzoites at 7, 14, 21, 30 dpi were collected. All the sera were subjected to antibody detection by Modified Agglutination Test (MAT), with the serum of mice with ME49-WT chronic infection as the positive control. F. Cysts in the brain homogenate smear were visualized under a fluorescence microscope (1000×) after staining with DAB-FITC antibody. Data are represented as mean ± SEM of three independent experiments. Statistical analysis was done by unpaired 2-tailed Student’s *t*-test. NS: no significant difference. Scale bar=10 μm (C), 20 μm (F).

Figure S2


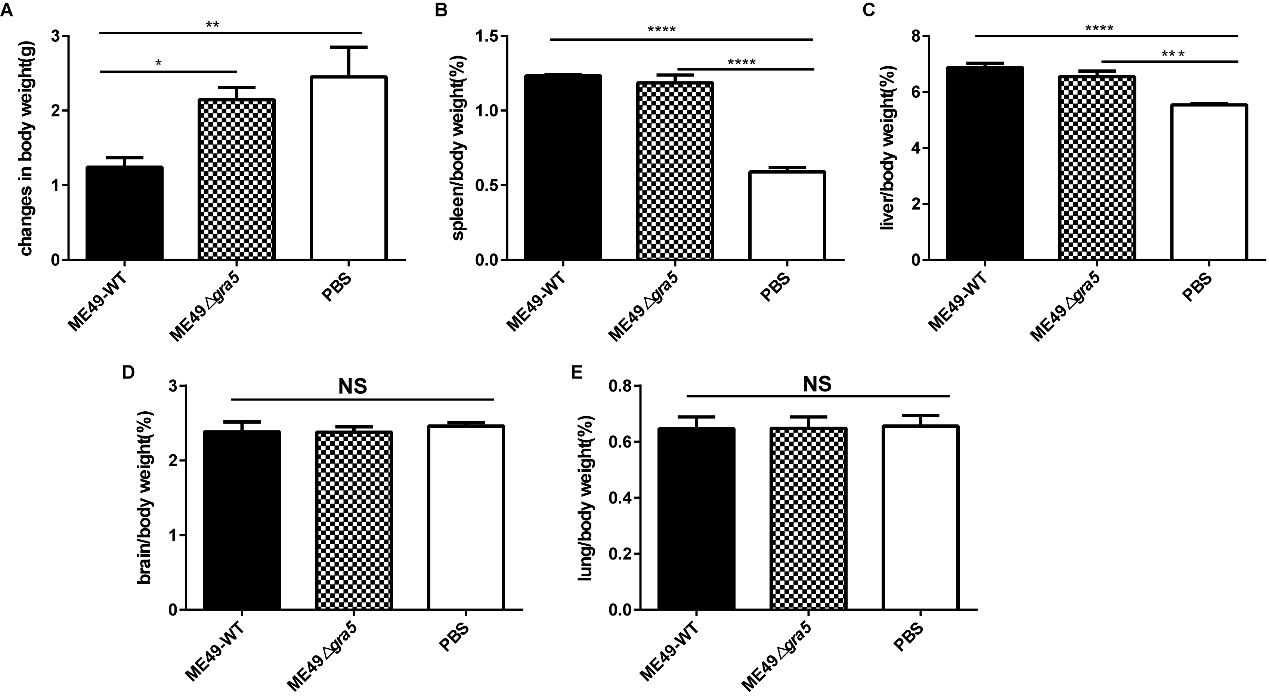


**Figure S2. Change of weight in bodies and organs of the mice infected with ME49 ME49∆*gra5,* or uninfected.** A-E. 6-8 weeks old female Balb/c mice were i.p. injected with 10^3^ ME49-WT or ME49∆*gra5* tachyzoites, and the same volume of PBS was inoculated as the control (n=5). Before infection and on the 7 dpi, each mouse was weighed, and the weight changes of each mouse were recorded (A). The mice were sacrificed and dissected at 7 dpi, and the weights of spleens (B), livers (C), brains (D), and lungs (E) were recorded and compared among the indicated groups of mice. Data are represented as mean ± SEM of three independent experiments. Statistical analysis was done by one-way ANOVA. NS: no significant difference. **p*＜0.05, ***p*＜0.01, ****p*＜0.001, *****p*＜0.0001.

Figure S3


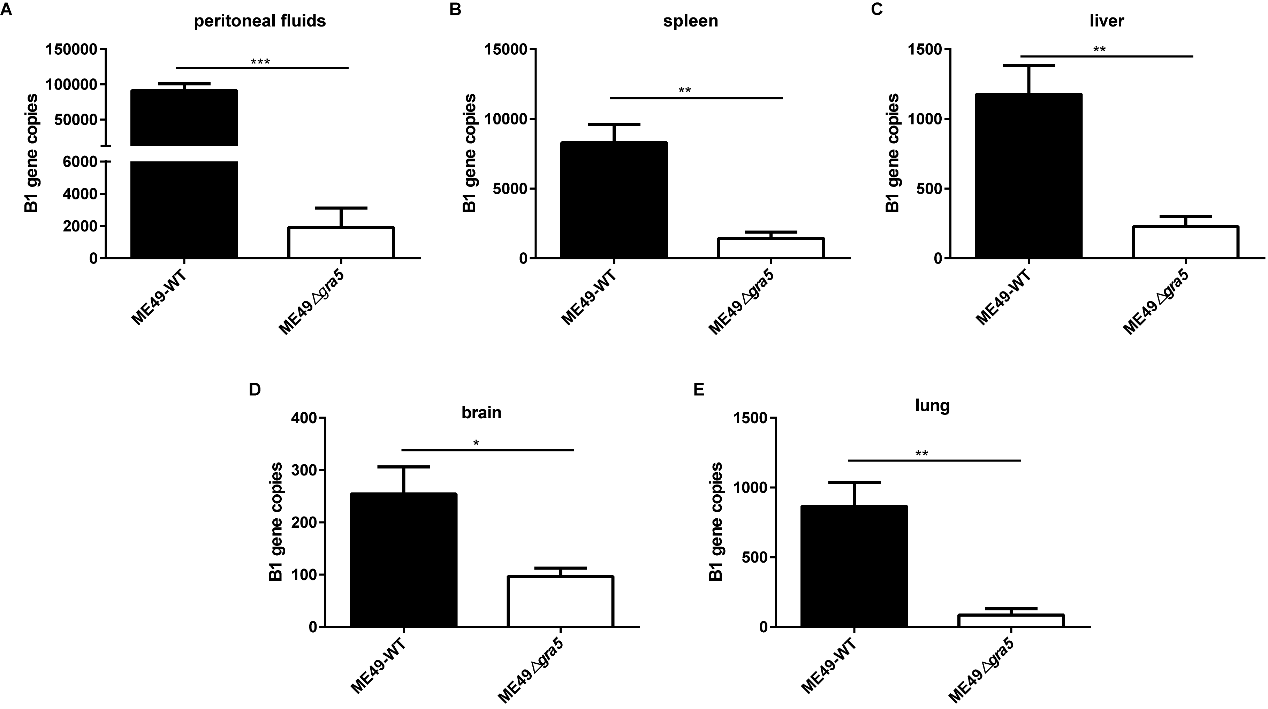


**Figure S3. Comparison of the parasitic burden of ME49 and ME49∆*gra5* in mice after infection.** A-E. 6-8 weeks old female Balb/c mice were i.p. injected with 10^3^ ME49-WT or ME49∆*gra5* tachyzoites (n=5). On 7 dpi, genomic DNA of peritoneal fluids, spleens, brains, and lungs were extracted. The B1 gene was detected by qPCR with the peritoneal fluids (A), spleen (B), liver (C), brain (D), and lung (E). Data are represented as mean ± SEM of three independent experiments. Statistical significance was assessed with a two-tailed unpaired Student’s *t*-test. **p*＜0.05, ***p*＜0.01, ****p*＜0.001.

Figure S4


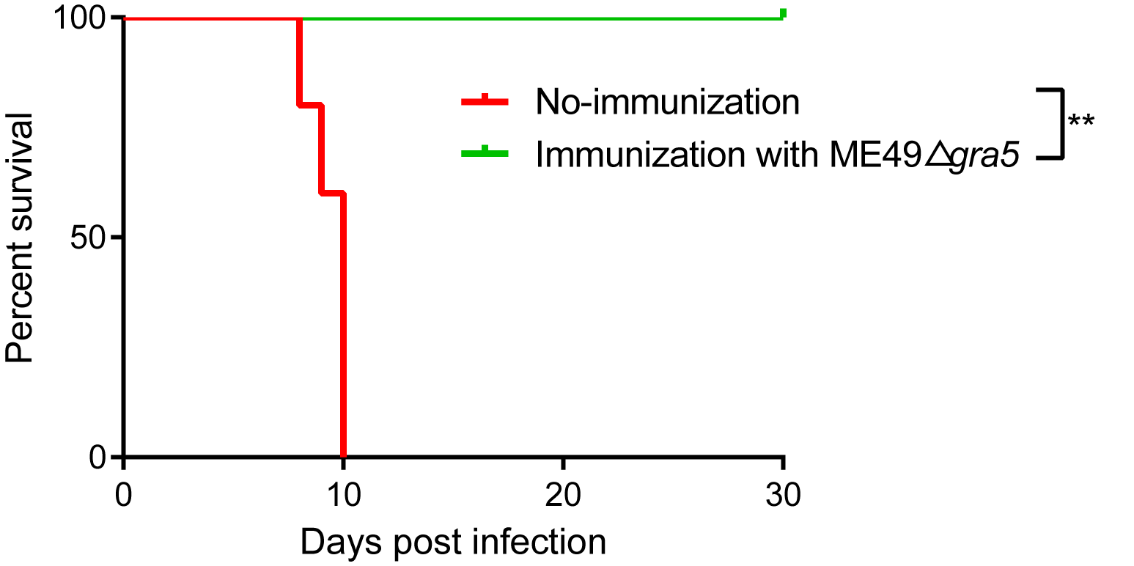


**Figure S4. ME49∆*gra5* tachyzoites vaccination protected mice from bradyzoites infection.** 6-8 weeks old female Balb/c mice were immunized with 10^3^ ME49∆*gra5* tachyzoites. After 30 days, mice were orally infected with 20 ME49 fresh brain cysts, and then the mice were monitored for another 30 days. The non-immunized mice were used as the control (n=5). Data are represented as mean ± SEM of three independent experiments. The survival curves were recorded for these two groups of mice. Statistical significance was assessed with log-rank (Mantel–Cox) test. ***p*＜0.01.

Figure S5


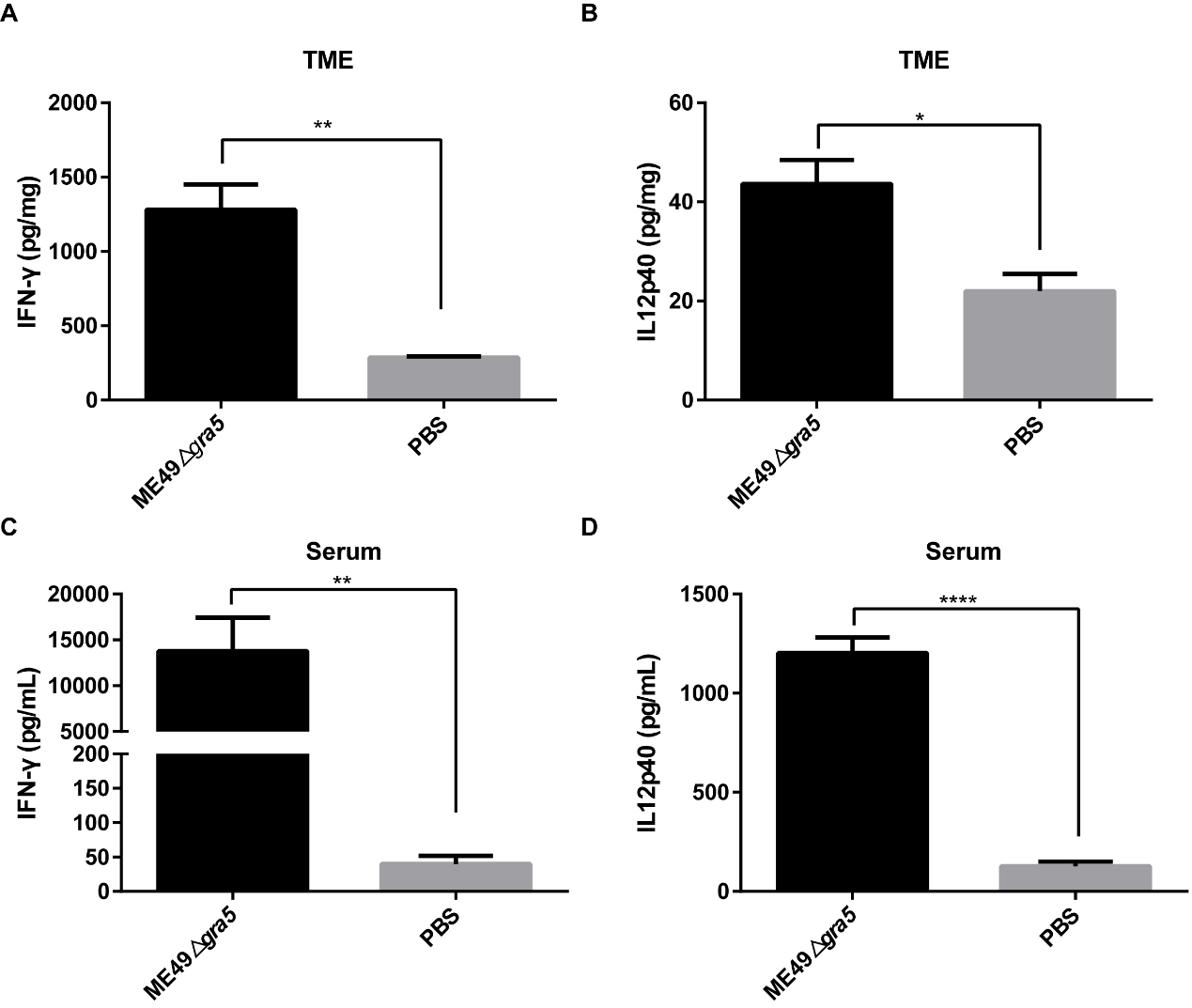


**Figure S5. ME49∆*gra5* intratumoral injection induced the production of IFN-γ and IL12 in both serum and TME.** A-D. The serum and tumors were collected on day 12 post 4T1 cell inoculation. The TME (A, B) and serum (C, D) were detected by ELISA to analyze the levels of IFN-γ (A, C), and IL12p40 (B, D). Data are represented as mean ± SEM of three independent experiments. Statistical significance was assessed with a two-tailed unpaired Student’s *t*-test. **p*＜0.05, ***p*＜0.01, *****p*＜0.0001.

Figure S6


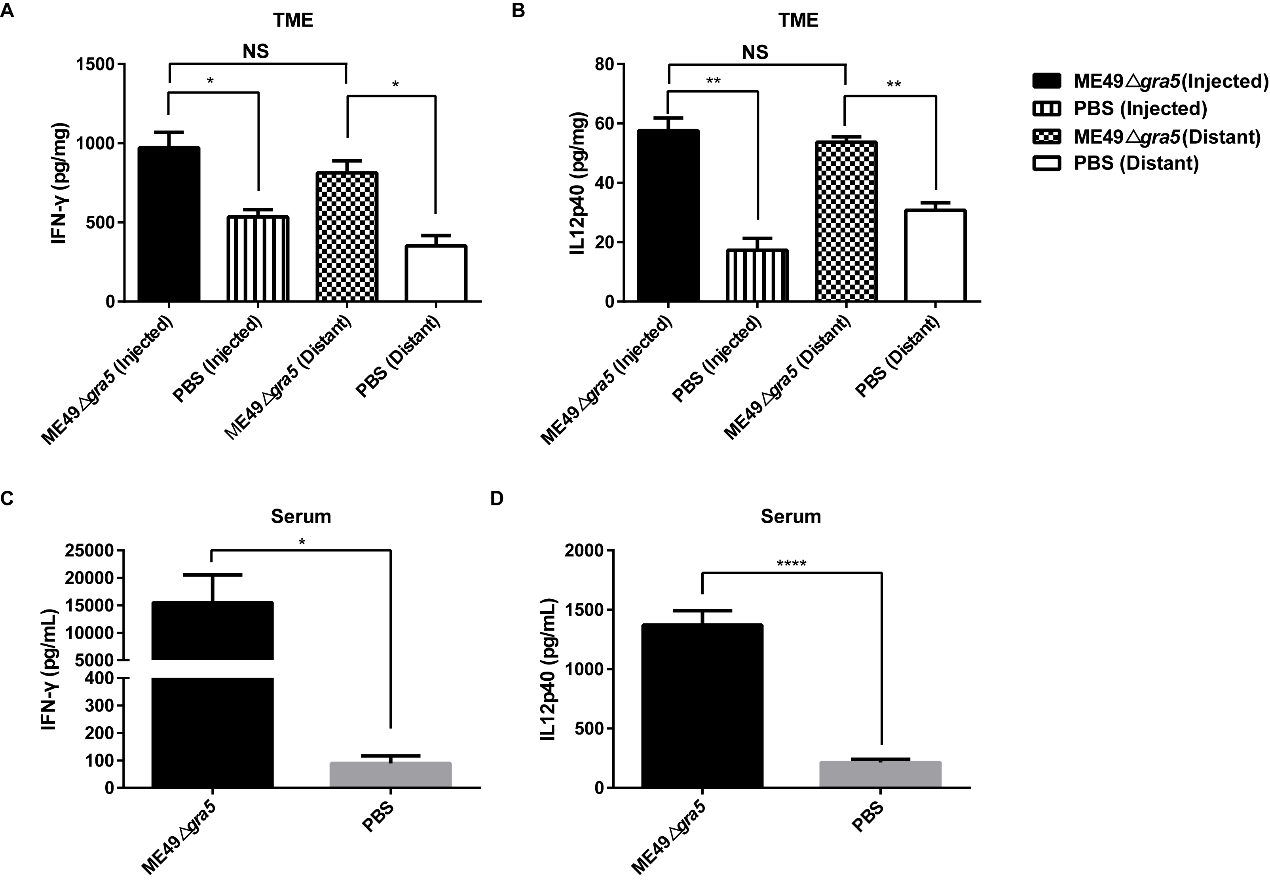


**Figure S6. ME49∆*gra5* stimulated non-injected distant tumors to generate IFN-γ and IL12.** A-D. The serum, injected tumors, and distant tumors were collected on day 12 post 4T1 cell inoculation. The injected and distant tumors (A, B), and serum (C, D) were detected by ELISA to analyze the levels of IFN-γ (A, C) and IL12p40 (B, D). Data are represented as mean ± SEM of three independent experiments. Statistical significance was assessed with a two-tailed unpaired Student’s *t*-test. **p*＜0.05, ***p*＜0.01, *****p*＜0.0001.
