# Supplementary figures and images for: Toxoplasma gondii gra5 deletion mutant protects hosts against Toxoplasma gondii infection and breast tumors
Source: Front Immunol. 2023 Jun 23;14:1173379. doi: 10.3389/fimmu.2023.1173379 (PMC10327641; doi:10.3389/fimmu.2023.1173379)

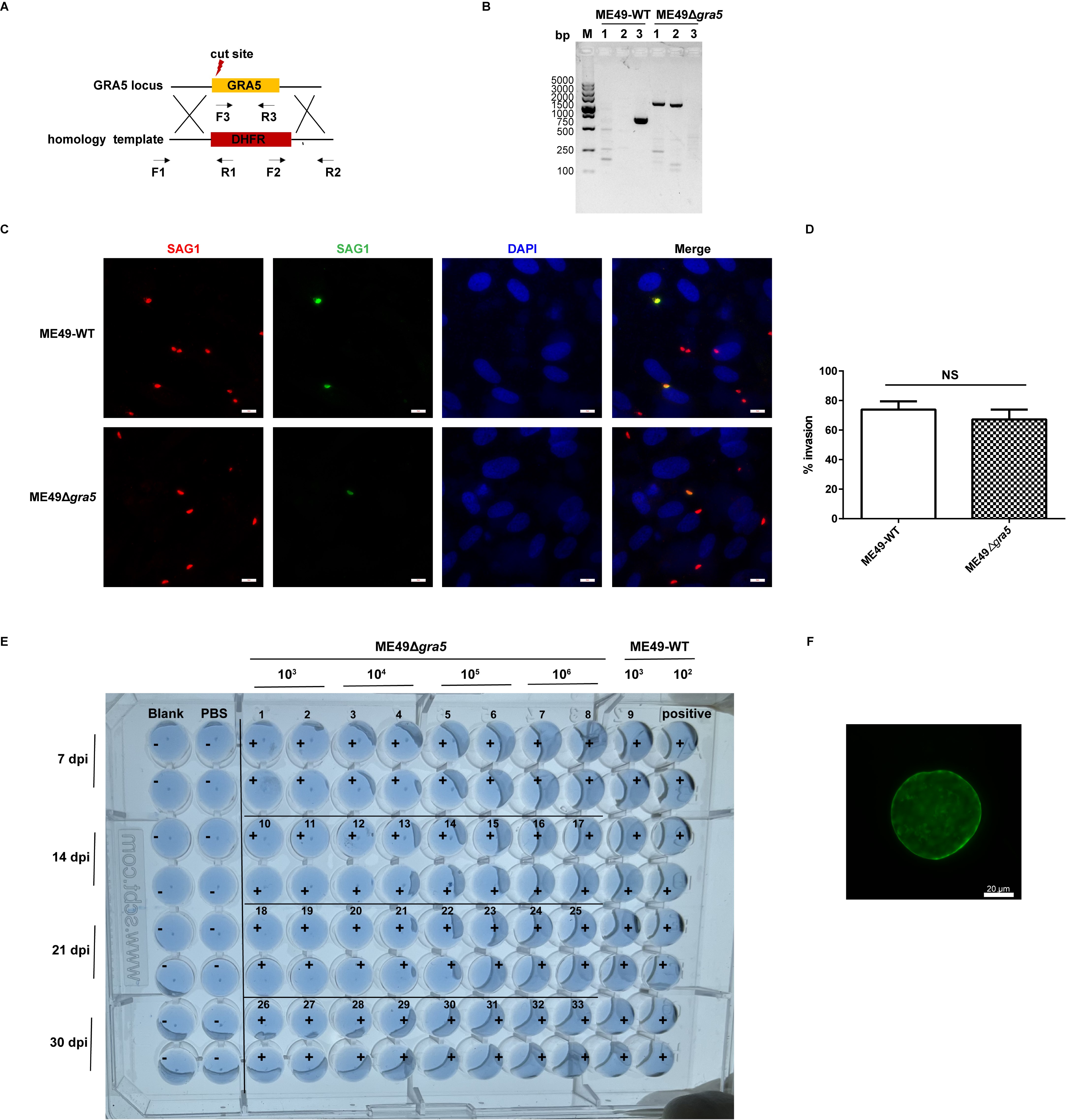

Supplement: Supplementary file 1 [file DataSheet_1.zip › supplementary information/Fig S1.jpg.jpg]

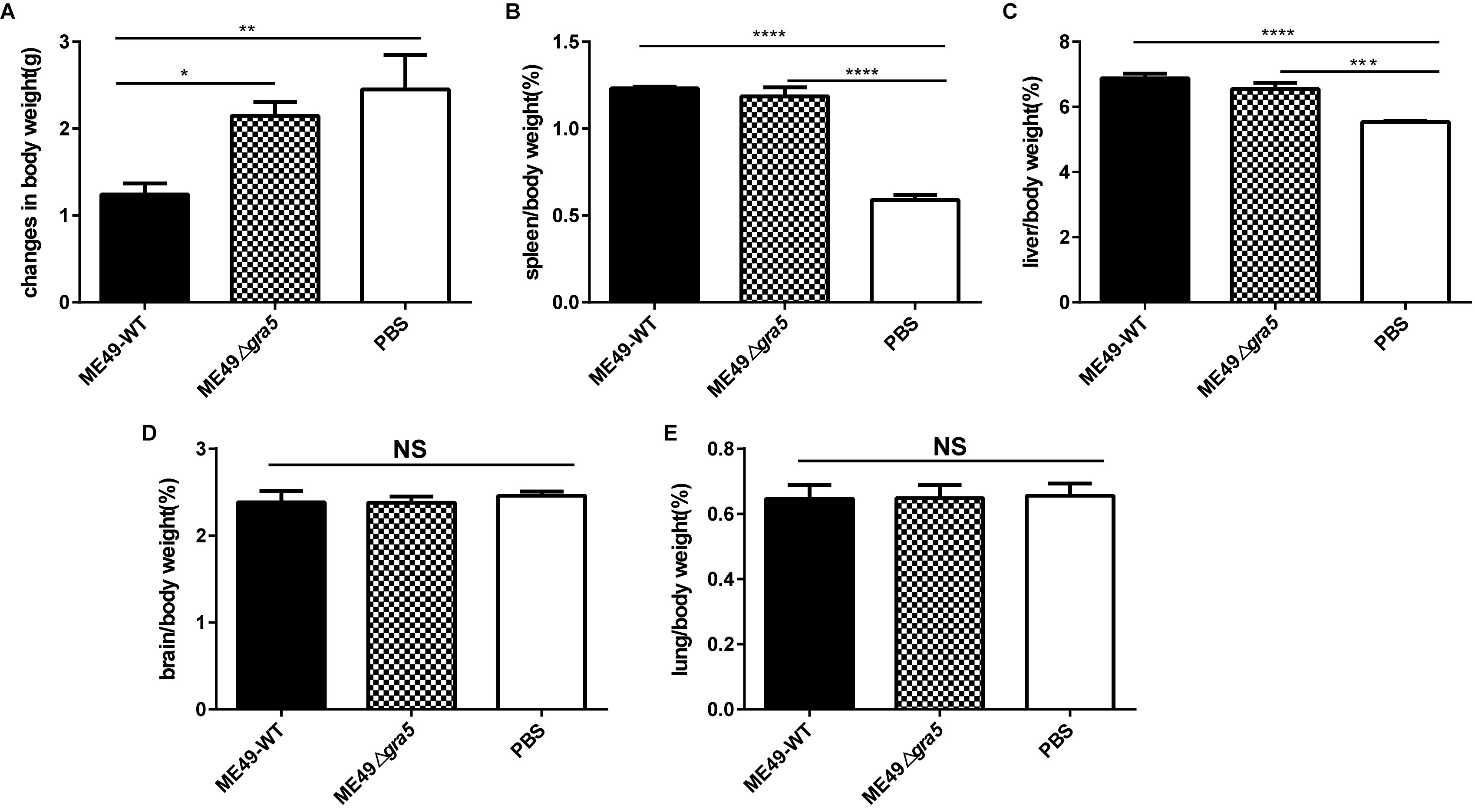

Supplement: Supplementary file 1 [file DataSheet_1.zip › supplementary information/Fig S2.jpg.jpg]

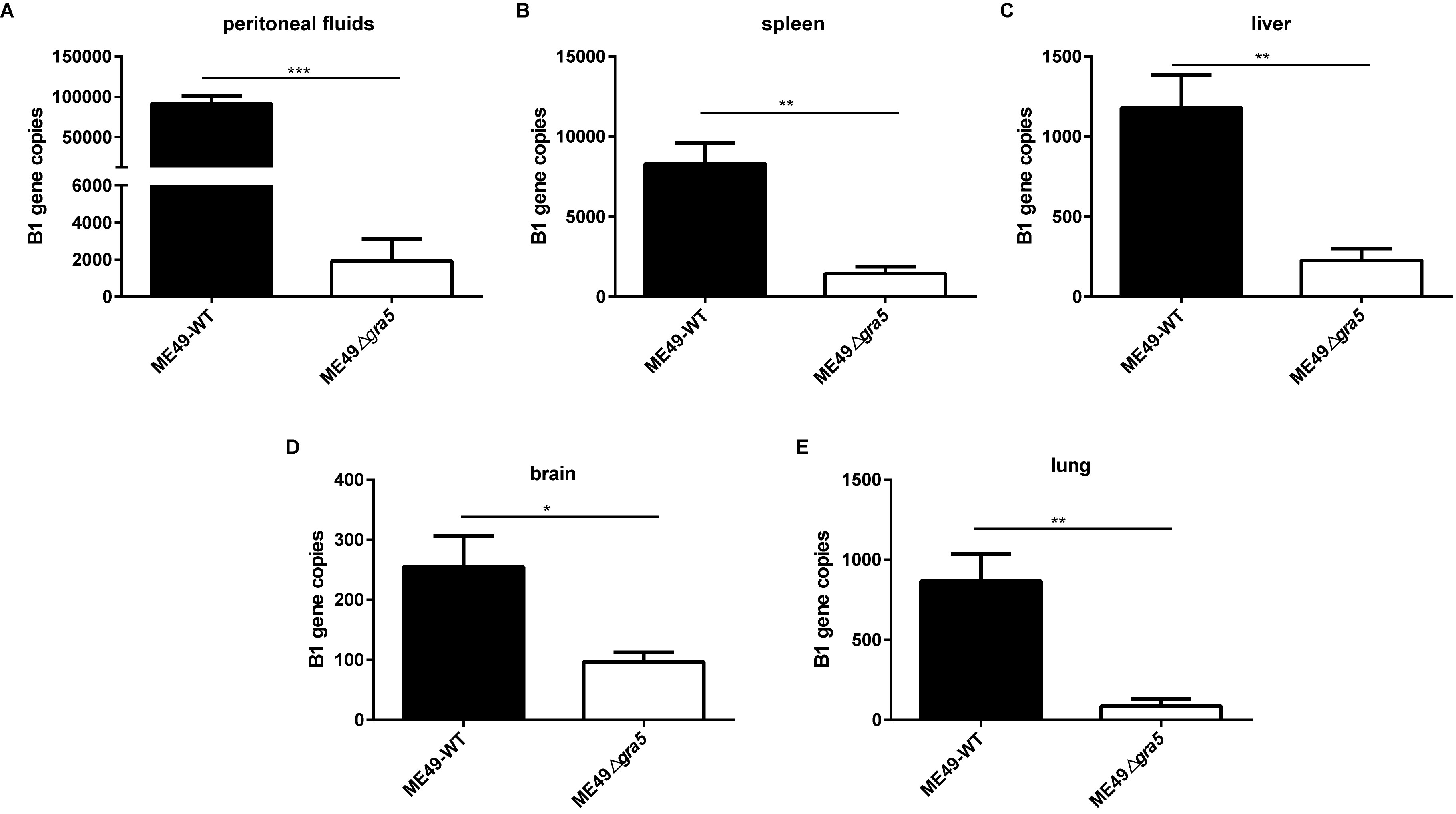

Supplement: Supplementary file 1 [file DataSheet_1.zip › supplementary information/Fig S3.jpg.jpg]

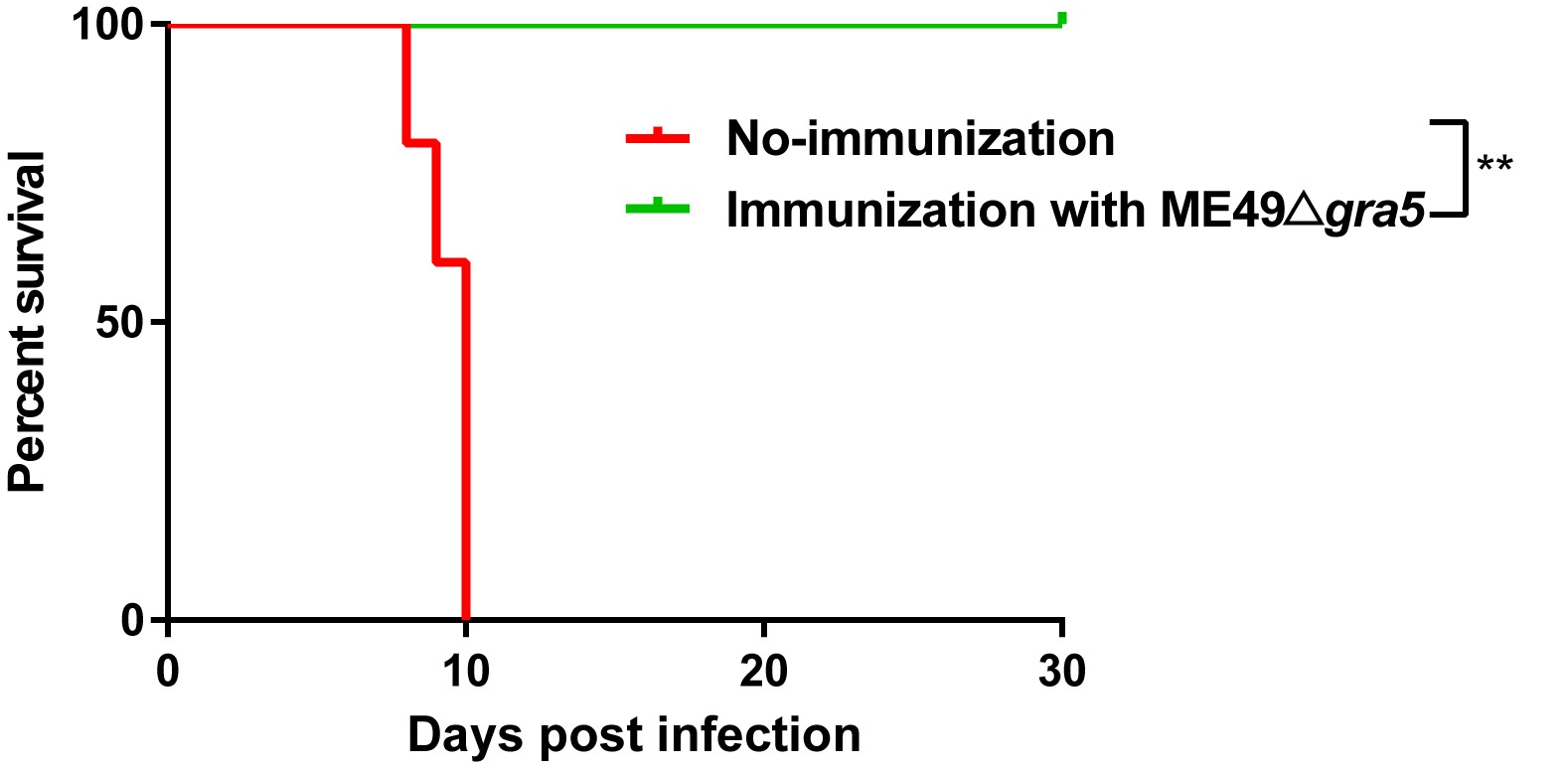

Supplement: Supplementary file 1 [file DataSheet_1.zip › supplementary information/Fig S4.jpg.jpg]

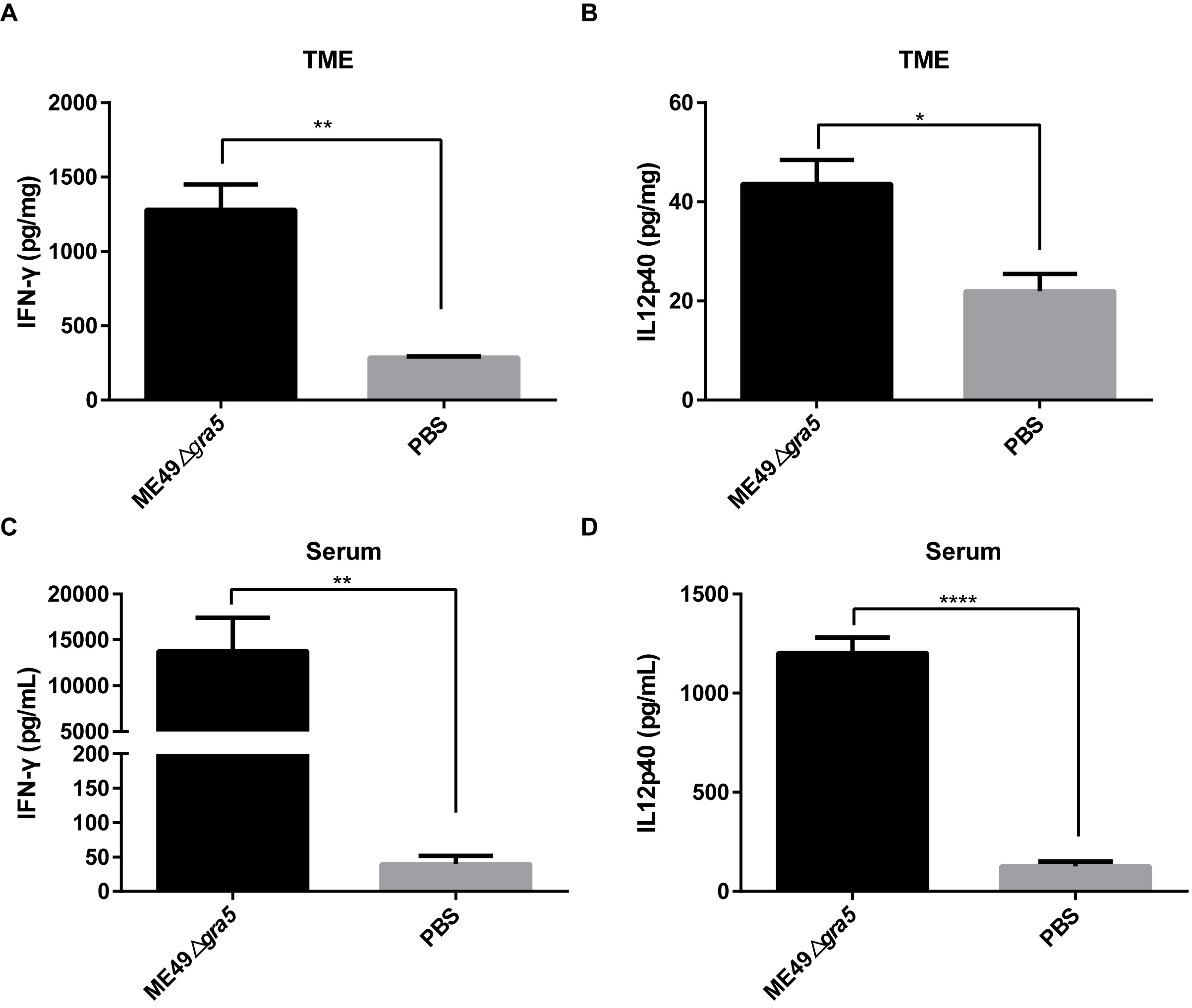

Supplement: Supplementary file 1 [file DataSheet_1.zip › supplementary information/Fig S5.jpg.jpg]

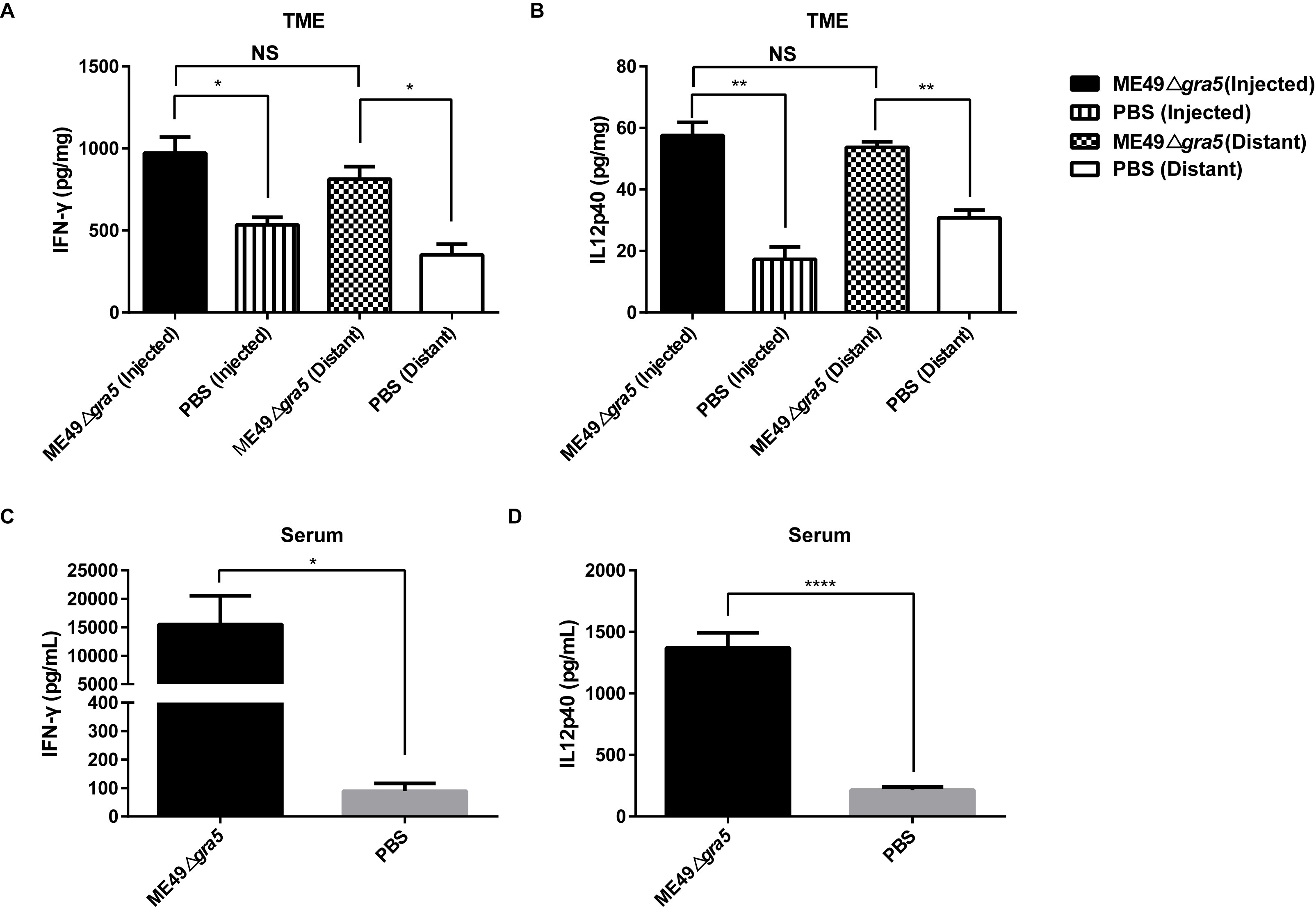

Supplement: Supplementary file 1 [file DataSheet_1.zip › supplementary information/Fig S6.jpg.jpg]
